# Artificial Intelligence (AI)-Aided Structure Optimization for Enhanced Gene Delivery: The Effect of the Polymer Component Distribution (PCD)

**DOI:** 10.1021/acsami.3c05010

**Published:** 2023-07-21

**Authors:** Yinghao Li, Zhonglei He, Sigen A, Xianqing Wang, Zishan Li, Melissa Johnson, Ruth Foley, Irene Lara Sáez, Jing Lyu, Wenxin Wang

**Affiliations:** † Research and Clinical Translation Center of Gene Medicine and Tissue Engineering, School of Public Health, Anhui University of Science and Technology, Huainan 232001, China; ‡ Charles Institute of Dermatology, School of Medicine, University College Dublin, Dublin D04 V1W8, Ireland; § Branca Bunús Ltd, NovaUCD Belfield Innovation Centre, Dublin D04 V2P1, Ireland; ⊥ School of Medicine, Anhui University of Science and Technology, Huainan 232001, China

**Keywords:** gene delivery vectors, poly(β-amino ester), polymer component distribution (PCD), pDNA delivery, machine learning, artificial intelligence

## Abstract

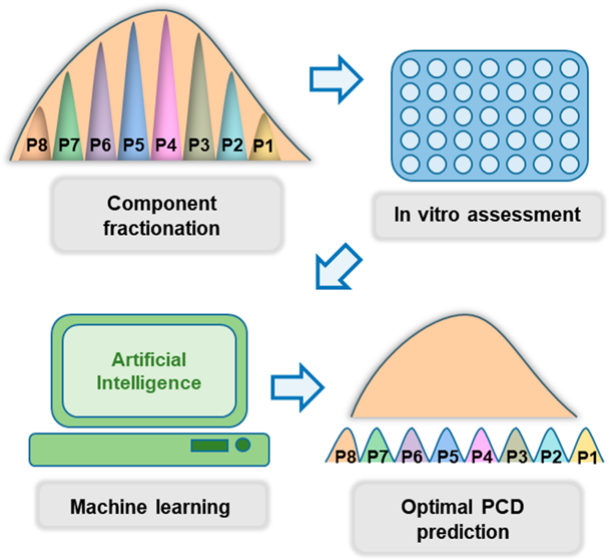

Gene therapy has emerged as a significant advancement in medicine in recent years. However, the development of effective gene delivery vectors, particularly polymer vectors, remains a significant challenge. Limited understanding of the internal structure of polymer vectors has hindered efforts to enhance their efficiency. This work focuses on investigating the impact of polymer structure on gene delivery, using the well-known polymeric vector poly(β-amino ester) (PAE) as a case study. For the first time, we revealed the distinct characteristics of individual polymer components and their synergistic effects–the appropriate combination of different components within a polymer (high MW and low MW components) on gene delivery. Additionally, artificial intelligence (AI) analysis was employed to decipher the relationship between the polymer component distribution (PCD) and gene transfection performance. Guided by this analysis, a series of highly efficient polymer vectors that outperform current commercial reagents such as jetPEI and Lipo3000 were developed, among which the transfection efficiency of the PAE-B1-based polyplex was approximately 1.5 times that of Lipo3000 and 2 times that of jetPEI in U251 cells.

## Introduction

Gene therapy is an innovative approach to treating human diseases by introducing genetic material into specific cells. It has gained significant importance in the medical field with the global market projected to reach $46.5 billion by 2030.^1^ Advances in molecular biology and biotechnology, along with the identification of disease-causing genes, offer potential treatments for conditions like hemophilia, muscular dystrophy, cystic fibrosis, cardiovascular diseases, neurological disorders, infectious diseases, wound healing, and cancer. The primary goals of gene therapy are to replace faulty genes, enhance natural proteins, modify gene expression, or produce targeted cytotoxic proteins.^2−4^ The success of gene therapy relies on the development of high-performance vectors, which are essential for delivering therapeutic genes to their intended destinations. Viral vectors, including adenoviral, lentiviral, retroviral, adeno-associated virus (AAV), and herpes simplex virus (HSV) vectors, are commonly utilized for gene delivery due to their high transgene expression. However, they have limitations such as immunogenicity, restricted packaging, and potential toxicity.^5,6^ Nonviral vectors, such as lipid- and polymer-based vectors, have emerged as promising alternatives. Lipid-based vectors form stable DNA nanoparticles, exhibit low immunogenicity, and can be customized for target specificity. Nonetheless, they may encounter challenges such as instability in serum and reduced transfection efficiency due to interactions with serum proteins. Some lipid-based vectors may also induce inflammation and cytotoxicity.^7,8^ In contrast, polymer-based vectors (e.g., poly(β-amino ester) (PAE), polyethylenimine (PEI), poly(l-lysine) (PLL), poly(dimethylaminoethyl methacrylate) (DMAEMA), etc.) employ cationic polymers to condense DNA into nanoparticles through electrostatic interaction, offering high stability, low immunogenicity, and the potential for improved target cell specificity. Polymer vectors are considered safe, accommodate large genes, and can be personalized. Ongoing optimization efforts aim to enhance their efficiency and safety for clinical applications.^9−11^

In light of the challenges associated with viral vectors and the advancements in nonviral gene delivery systems, poly(β-amino esters) (PAEs) have emerged as highly promising and versatile polymer vectors for efficient gene delivery since they were initially developed by Langer’s group in 2000.^12^ PAEs offer advantages such as easy modulation of solubility, charge, and reactivity through protonation and deprotonation of ester and amine groups. PAEs’ biodegradability, efficient endosomal escape, payload unpacking ability, and customizable physicochemical properties make them stand out among the polymer vectors. Up to now, thousands of PAE vectors have been developed, highlighting their significance in the field of gene therapy.^13−15^ Despite extensive research and the development of numerous PAE vectors, finding candidates with an efficiency comparable to viruses remains challenging. The field of polymeric gene carriers has reached a bottleneck, necessitating further advancements to overcome this hurdle.

To break through this bottleneck, it is imperative to understand the unique characteristics that distinguish polymers from other vectors. Most obviously, unlike viruses and lipids which have defined chemical structures, the synthetic polymer vectors (e.g., PAE, PEI, PLL, DMAEMA, etc.) are a mixture of components with different molecular weights (MWs).^11,16,17^ The different polymer components should, in principle, play different roles and synergistically work together in the complex process of gene delivery. For example, previous studies showed that polymer components with high MWs can act as steel bars, making the polymer structure stable for protecting the DNA core.^18,19^ While considering that the high MW components are inefficient in shielding the DNA charge due to their large steric hindrance, the mobile small MW components can contribute to adequately shield the negative residue charges on phosphate groups, helping the DNA to coil and fold further.^20^ Moreover, other MW components may aid in polyplex cellular uptake, endosomal escape, and nuclear pore entry.^21,22^ It is conceivable that polymer components with different structures favor the specific interactions between the polymer and DNA mentioned earlier. These polymer components with diverse structures combine to form polyplexes, ultimately achieving efficient transfection. Therefore, to optimize the overall gene transfection process, it is essential to determine the optimal combination ratio of different polymer components in the overall polymeric vectors, that is, the polymer component distribution (PCD). Unfortunately, so far, our understanding of the relationship between the intrinsic components of polymers and their gene delivery performance is still limited. In contrast, most of the studies on polymeric vectors have been focused on the general polymer properties, e.g., overall topology, chemical compositions, average MW, etc.^11,13,16,17^

This work uses a highly versatile gene delivery vector PAE as the model to study, for the first time, the effect of PCD on transfection. First, various components of the PAEs were isolated, and the individual behavior of each component as well as their synergistic behavior in gene transfection were investigated and compared. Second, a library of PAEs with different PCDs was prepared and tested for transfection. Based on these results, a machine learning–deep learning approach was conducted to establish the polymer PCD-transfection performance relationship. Finally, a highly efficient PAE vector with optimized PCD was predicted by the trained model, and its robust transfection capability was verified further, which far surpassed the well-known commercial reagents jetPEI and Lipo3000 (Scheme S1).

## Results and Discussion

To understand the relationship between PCD and polyplex transfection performance, a linear PAE (LPAE) was investigated in terms of the following questions: (1) What are the different polymer components? (2) Can variations in PCD affect the transfection performance? (3) How can PCD influence transgene expression? (4) Can these concepts lead to high-performance polymer vectors generated by optimizing PCD?

### What are Different Polymer Components?

**Scheme 1:**
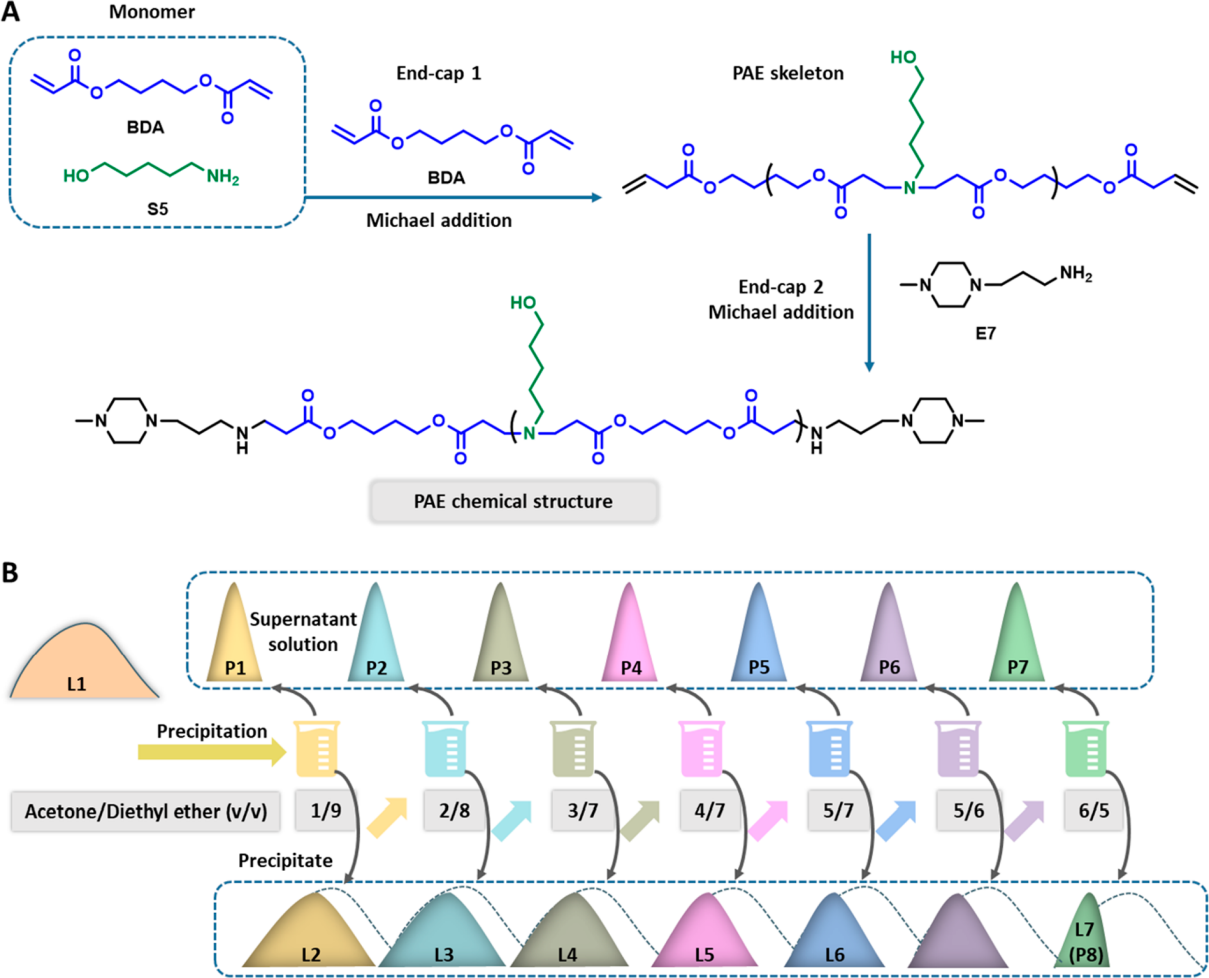
Schematic Illustration of the LPAE Polymerization and Fractionation Processes (A) LPAE L1 was synthesized *via* the Michael addition approach from 1,4-butanediol diacrylate (BDA), 5-amino-1-pentanol (S5), and 1-(3-aminopropyl)-4-methylpiperazine (E7, end-capping reagent). The LPAE backbone was end-capped first by BDA and then by E7 before purification. (B) LPAE L1 was fractionated into P1–P8, respectively, by precipitating into a solvent mixture of acetone/diethyl ether (v/v = 1/9 to 6/5). The components P1–P7 were collected from the supernatant solution. The residual polymers L2–L7 were collected from the precipitates. L7 was also named as P8, which represents the component of highest molecular weight.

One batch of LPAE (L1) was synthesized via a facile Michael addition approach as described in Scheme 1A. The widely used PAE monomer combination of 1,4-butanediol diacrylate (BDA) and 5-amino-1-pentanol (S5) was selected to construct the polymer backbone.^23,24^ The high-performance amine, 1-(3-aminopropyl)-4-methylpiperazine (E7),^25,26^ was used as the end-capping reagent. To ensure consistency of the type of terminated groups in different components of L1, BDA was added to eliminate the S5 terminal amines prior to E7 end-capping. The final structure was thoroughly verified by gel permeation chromatography (GPC/SEC) and nuclear magnetic resonance (NMR) (Figures S1–S7, Table 1).

**Table 1:** SEC Analysis Results of Different Components P1 to P8 Obtained from Fractionation

Polymer	*M*_p,SEC_^a^ (Da)	*M*_n,SEC_^b^ (Da)	*M*_w,SEC_^c^( Da)	*Đ*^d^
L1	4365	3757	10 524	2.80
P1	2138	1894	2225	1.17
P2	2727	2414	2881	1.19
P3	3652	3181	3880	1.21
P4	5052	4177	5271	1.26
P5	7579	5429	8070	1.48
P6	10 484	7351	10 784	1.46
P7	14 982	11 341	15 837	1.39
P8	23 984	11 765	28 717	2.44

a *M*_p,SEC_ is the molecular weight of the peak maxima acquired from SEC.

b *M*_n,SEC_ is the number-average molecular weight acquired from SEC.

c *M*_w,SEC_ is the weight-average molecular weight acquired from SEC.

d *Đ* is the polydispersity index acquired from SEC.

**Figure 1:**
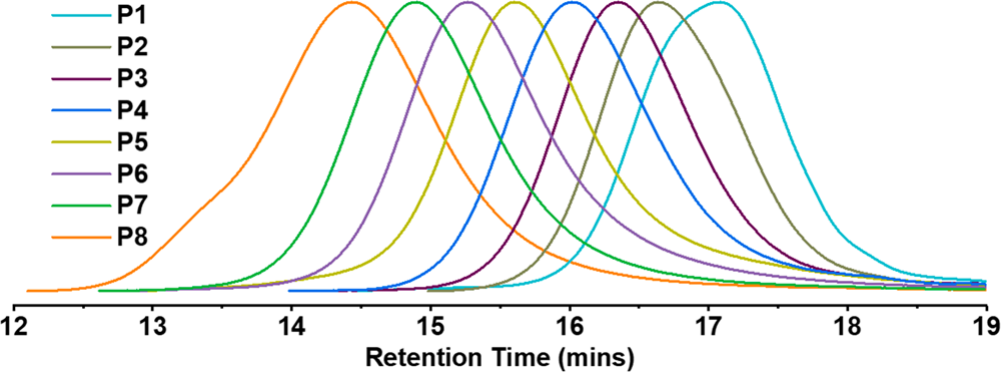
SEC traces of components P1 to P8.

Subsequently, as depicted in Scheme 1B, L1 was fractionated into eight components, P1–P8, and eight residual polymers named L1–L8, respectively, were obtained. In detail, L1 was first dissolved in acetone with a concentration of 100 mg/mL, and the solution was slowly added into a solvent mixture of acetone/diethyl ether (v/v = 1/9) under gentle agitation at room temperature. The precipitate was then collected as residue polymer L2, and the component P1 was obtained by evaporating the remaining supernatant solution. A similar process for L2 to L7 produced polymer components P2 to P8 by using solvent mixtures with increasing acetone content (acetone/diethyl ether = 2/8 to 6/5) (Scheme 1B). Here, L7 was also named P8, representing the highest MW components of L1. Table 1 and Figure 1 show the SEC characterization results for each individual component P1–P8 fractionated from L1, where parallel movements of the MWs from low to high (*M*_n,SEC_ = 1894 to 11765 Da) for P1 to P8 can be clearly observed.

### Can PCD Affect Transfection Performance?

**Figure 2:**
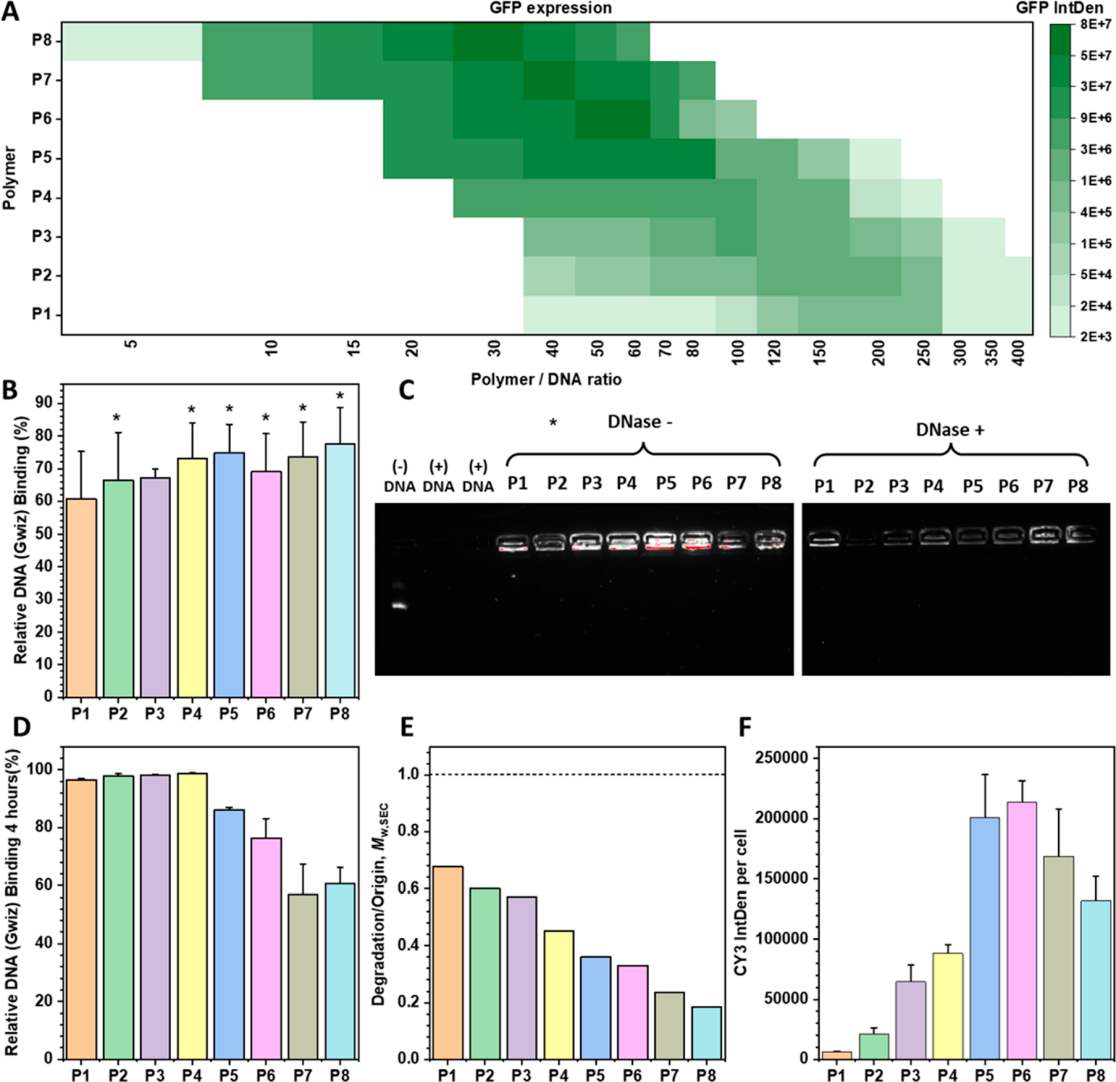
Biophysical characterization of polymer components. (A) GFP expression of different PAE components (P1–P8, with *M*_w,SEC_ values ranging from 2.2 to 28.5 kDa) at different polymer/DNA w/w ratios. (B) The relative DNA binding efficiencies. One-way ANOVA, mean ± SD; data points marked with asterisks (*) are statistically significant relative to the P1 group. *P < 0.05, superior DNA binding efficiencies compared with that of P1. (C) DNA condensation and protection properties of polymer components under their optimal polymer/DNA w/w ratio. For DNA protection study, DNase I was added with polyplexes under the concentration of 10 U DNase I/1 μg DNA (+). Naked NDA with and without DNase I were used as control. (D) DNA protection efficiencies under acid condition (post 4 h incubation at 37 °C in pH 4.8 sodium acetate buffer). (E) Polymer components degradation behavior (post 4 h incubation at 37 °C in pH 4.8 sodium acetate buffer). Degradation/Origin of components P1–P8 was calculated as the ratio of their *M*_w,SEC_ before and after degradation (Figure S8). (F) Cellular uptake performances of P1–P8 under their optimal polymer/DNA w/w ratio.

Based on the above understanding of polymer component information, to determine whether the PCD has an effect on the PAE gene transfection performance, the transfection behavior of these different components P1–P8 (Figure 1, Table 1) was first evaluated by delivering a gWiz-GFP plasmid at different polymer/DNA ratios in a common human brain tumor cell line (U251-MG). Surprisingly, as shown in Figure 2A, the working doses of different components exhibited a clear MW dependence. Specifically, the higher MW components achieved optimal results at lower polymer/DNA ratios, while the lower MW components required a higher polymer dose to achieve better GFP expression. Moreover, it is worth noting that with the increase of the MWs of P1–P8, their transfection efficiency at the optimal polymer/DNA ratios first increased and then decreased with the maximum transfection efficiency reached at P6 (polymer/DNA ratio = 60:1).

The next step was to identify how each particular process during gene transfection was affected, leading to the above different transfection performances of P1–P8. The performance of several key transfection processes, DNA packaging, cellular uptake, and DNA protection, was investigated at each component’s optimal polymer/DNA ratio. According to Figure 2B, in terms of the DNA packaging process, as the MWs of different components increased, the DNA binding efficiency only showed a slight increase from 60% to 76%. This contrasted with the significant differences in the transfection performance between different components. The DNA condensation and protection abilities of P1–P8 were assessed by using gel electrophoresis (Figure 2C). Remarkably, even though P1 has the lowest *M*_w,SEC_ of 2225 Da, it achieved complete DNA condensation without any DNA migration. The DNA protection capacity of P1–P8 against degradation by the DNase I enzyme was also investigated (DNase + ). It was observed that DNA without polymer protection underwent complete degradation, while the high MW components exhibited an effective DNA protection capability, retaining most DNA in the polyplexes. Notably, the lowest MW component P1 also demonstrated a significant DNA protection capability against enzymes. The DNA binding efficiency under acidic conditions was evaluated as another indicator of DNA protection in the endosome (Figure 2D). After incubation at 37 °C, polyplexes formed by lower MW components (P1–P4) achieved 100% encapsulation capacity, while the encapsulation of polyplexes formed by higher MW components (P5–P8) exhibited varying degrees of decrease. This suggests that smaller MW components require more time to form highly compressed and stable polyplex structures. In contrast, higher MW components rapidly condensed DNA, but their resulting polyplexes were more susceptible to degradation and depackaging. The degradation profile of P1 to P8 confirmed this observation (Figure 2E and Figure S8), that is, the polymer components with higher MWs were more prone to degrade under acidic conditions, aligning with the decrease in DNA binding efficiencies observed in Figure 2D. In terms of the cellular uptake process of the polyplexes of P1–P8, notably, a consistent trend of changes with their transfection performance was observed (Figure 2A). As can be clearly seen in Figure 2F, from P1 to P8, the cellular uptake first increased and then decreased, and the optimal value appeared at P6. These results indicate that the use of different polymer components can indeed affect several gene transfection processes at different stages. In particular, the cellular uptake is the key process that determined the transfection performance of different components.

**Figure 3:**
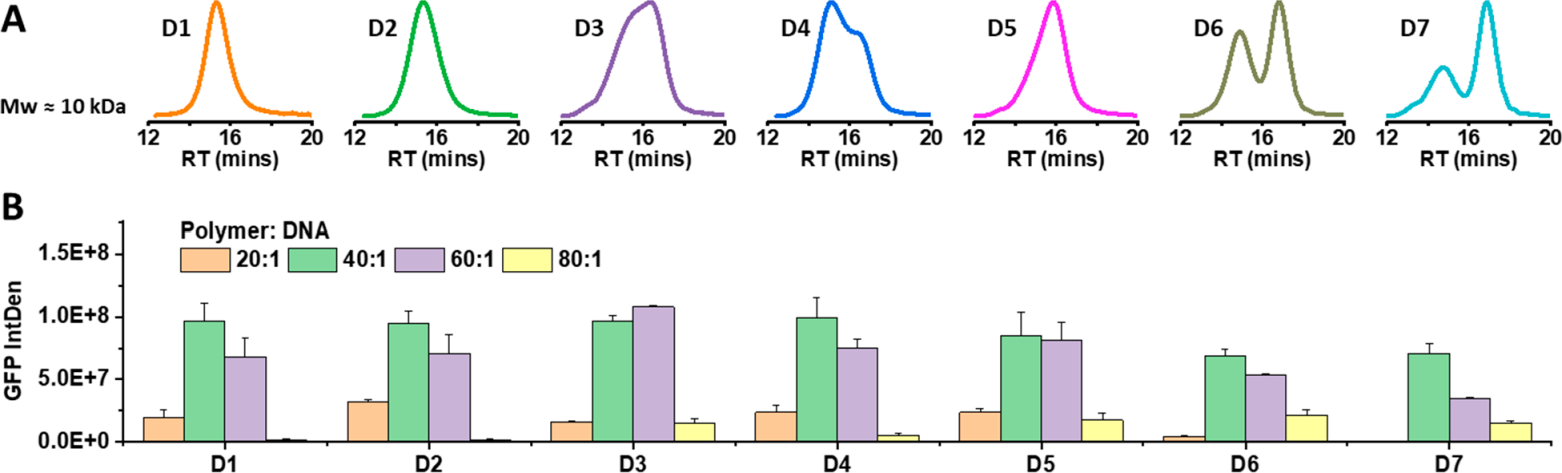
Characterization and assessment of reconstituted PAEs with different polymer component distributions (PCD). (A) SEC traces of polymers D1-D7 with similar *M*_w,SEC_ (10 kDa) values but various PCDs (Table 2). D1–D7 were prepared by mixing polymer components P1–P8 with different weight ratios, as shown in Table 2. (B) Transfection results of D1–D7 polyplex with U251-MG cells after 48 h at polymer/DNA w/w ratios of 20:1 to 80:1.

Based on the above insights on the effect of each individual component on transfection, P1 to P8 were mixed by different weight ratios to reconstitute D1–D7 (Table 2, Figure 3A) to further investigate if the polymer PCD affects the transfection performance. As displayed in Table 2 and Figure 3, polymers D1-D7 with similar *M*_w,SEC_ (10 ± 0.5 kDa) but different PCDs (unimodal/multimodal, *Đ* ranges from 2.2 to 4.3) were reconstituted and evaluated. The transfection results in Figure 3B clearly showed that even with similar MWs or *Đ*, varying the PCDs of D1–D7 resulted in different transfection behavior. For example, while PAEs D2 and D5 have similar *M*_w,SEC_ and *Đ*, D5 contains more low MW components (P4, Table 2) and achieved better transfection efficiency at a broader polymer dose (40–60:1). Moreover, the molecular weight distributions (MWDs) of D3, D4, D6, and D7 altered from unimodal to multimodal as intermediate MW components decreased (Table 2), and their overall transfection capability likewise gradually decreased. These results demonstrate that the PCD indeed has an effect on the transfection behavior of the polymeric vectors, which cannot be simply described by the average properties of MW and/or *Đ*.

**Table 2:** SEC Characterization Results and Components Weight Ratio of Reconstituted Polymers D1 to D7

Polymer	Component ratio P1:P2:P3:P4:P5:P6:P7:P8	*M*_p,SEC_ (Da)	*M*_n,SEC_ (Da)	*M*_w,SEC_ (Da)	*Đ*
D1	0:0:0:0:0:40:0:0	10 484	7351	10 784	1.45
D2	0:0:0:5:7:20:8:0	9825	5240	11 548	2.20
D3	2:5:5:6:6:6:5:5	3712	3571	11 049	3.09
D4	3:3:4:4:4:5:17:0	12 133	3910	10 808	2.76
D5	0:0:0:18:7:6:5:4	5846	4275	11 053	2.58
D6	10:10:0:0:0:0:18:2	2356	2990	10 718	3.58
D7	18:8:0:0:0:0:6:8	2208	2699	11 723	4.34

### How Does PCD Affect Transfection?

**Figure 4:**
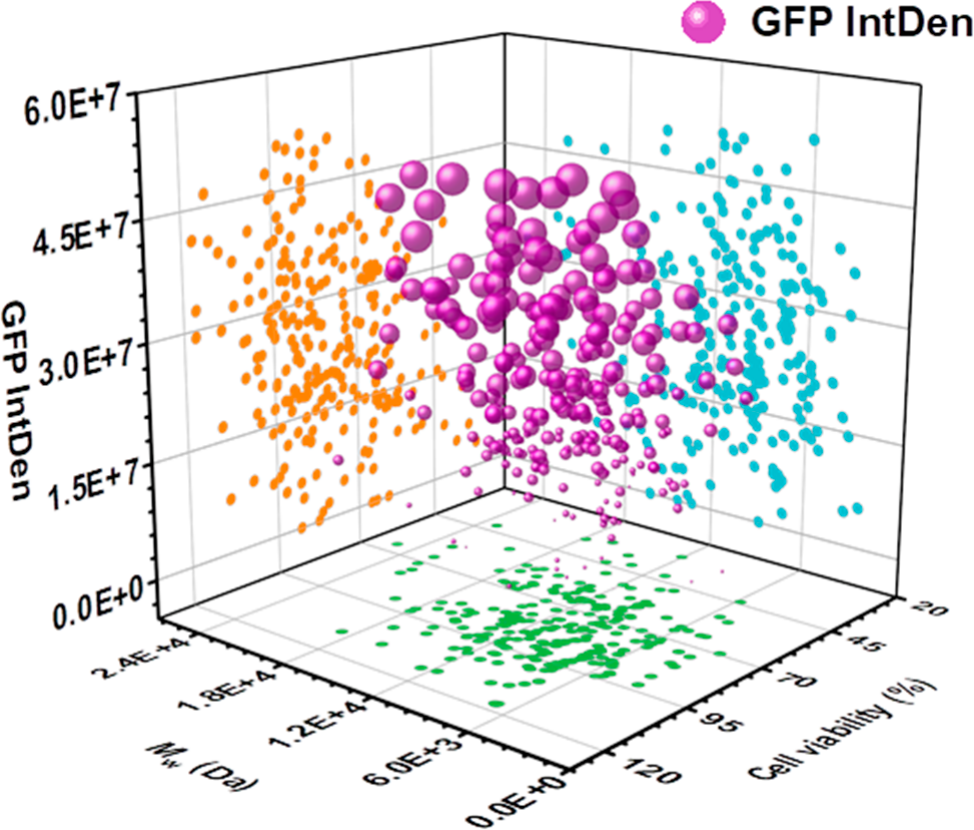
In vitro assessments of the GFP expression and cell viability of reconstituted PAEs with different *M*_w,SEC_ values on U251-MG cells. The reconstituted PAEs were reconstituted by mixing P1–P8 with different weight ratios (Table S1 and Table S2). The relationship between GFP expression and cell viability is visually represented by the orange dots. Similarly, the green dots indicate the relationship between the polymer molecular weight and cell viability. On the other hand, the relationship between GFP expression and polymer molecular weight is depicted by the blue dots.

Further, to interpret how PCD affects the transfection performance, a series of different PAE polymers were constructed by mixing the polymer components P1–P8 using different weight ratios (Table S1, Table S2). The transfection and cytotoxicity evaluation results of these reconstituted polymers with U251-MG cells are shown in Figure 4. Previous studies have reported that moderate polymer MWs (10–15 kDa) with the highest biocompatibility are optimal for achieving efficient and successful transfection.^27,28^ However, interestingly, according to Figure 4, this previous conclusion on the influence of MWs and cytotoxicity on transfection efficiency is not applicable. Specifically, within both the high and low MW ranges, many polymers with high cytotoxicity achieved efficient transfection, while in contrast, many reconstituted PAEs with desired MWs (within the range of 10–15 kDa) and low cytotoxicity did not achieve efficient gene delivery. These results further confirmed that the differences in the PCDs of these polymers significantly affected their gene transfection performance. Unfortunately, from the experimental analysis in Figure 4, the factors governing this influence are currently difficult to describe further.

In view of the above experimental analysis results, the effect of different components on gene delivery is complex and far beyond the simple cumulative effect of each individual component. It is thus necessary to find a powerful methodology to aid the investigation of the influence that the PCD of polymers exerts on their transfection performance. Recently, enabled by modern algorithms and high-performance computing, artificial intelligence (AI) (machine learning) has been progressively applied in polymer science^29^ for the design of gene delivery vectors.^30−32^ Herein, to help reveal how the different PCDs affect the transfection performance, machine learning was also conducted (Scheme S2), where six machine learning models (Support Vector Machine (SVM), k-Nearest Neighbors (KNN), Decision Tree (DT), Extreme Tree (ET), Random Forest (RF), and eXtreme Gradient Boosting (XGBoost)) were compared. After a 10-fold cross validation, the eXtreme Gradient Boosting (XGBoost)^33^ model achieved the lowest values of MAPE, MAE, MSE, RMSE, and the highest mean *R*^2^ (closest to 1) (Table S3, Figure S9), demonstrating its best performance among the six models. Therefore, XGBoost was then chosen to decipher the relationship between polymer components and transfection performance in the following text. Further, a grid search (GS) technique was used to tune the hyperparameters of the XGBoost model and achieved *R*^2^ = 0.8908 of the validation group. At the end, the machine learning interpretability method—SHapley Additive exPlanations (SHAP)^34^—was employed to compute the relative importance of different factors on transfection.

**Figure 5:**
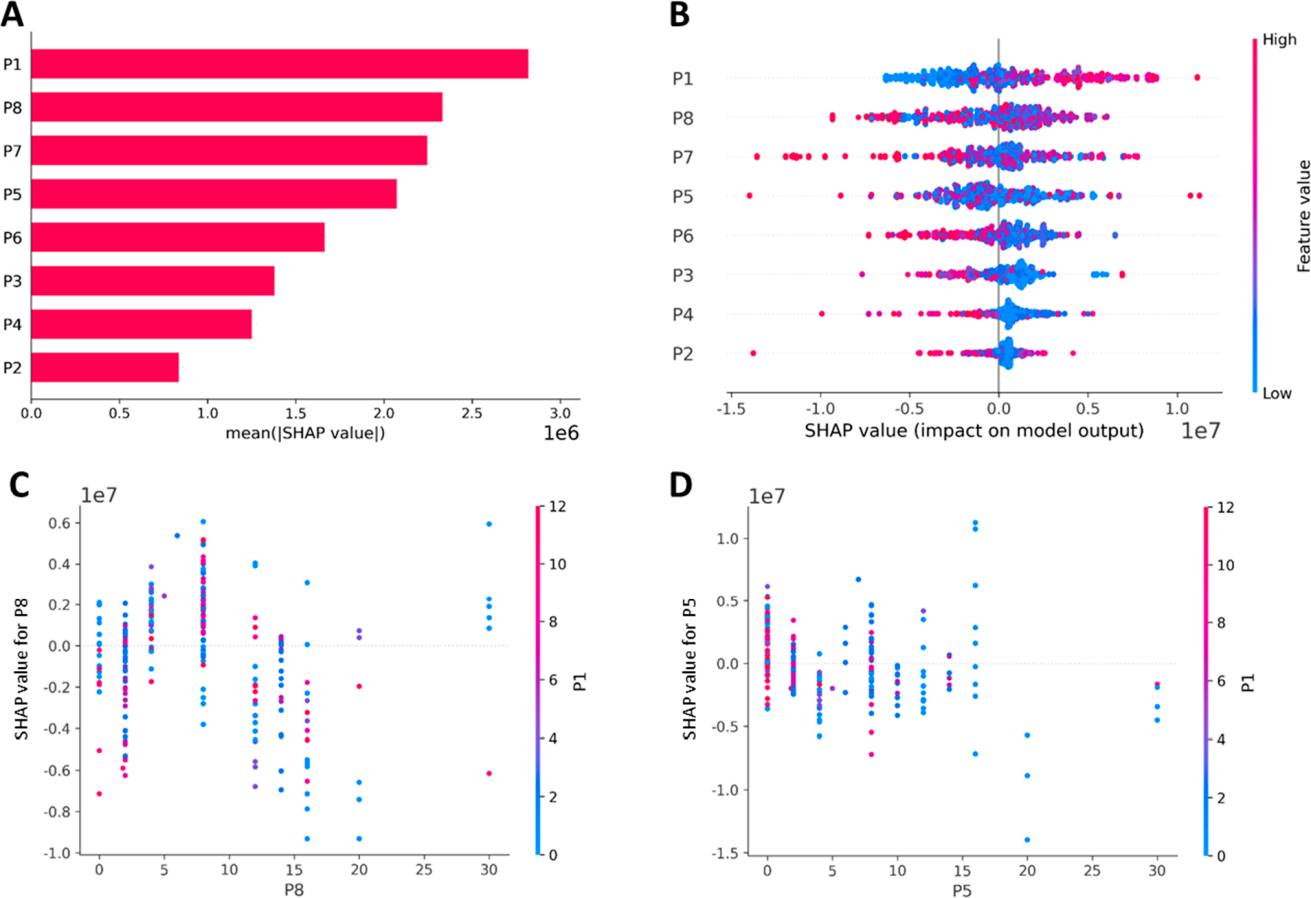
AI-aided relationship studies between the polymer component distribution and transfection performance. SHAP value was calculated based on game theory and local explanations, which can reflect the contribution of each feature (component ratio in reconstituted polymer) on model output (transfection performance). (A) Global importance (measured as their mean absolute SHAP value) of different components on transfection. Higher SHAP absolute values correlate to higher impact on the transfection performance. (B) SHAP values for different components related to transgene expression. The color bar of feature value corresponds to the normalized extent of different component (where low extent = blue; moderate extent = purple; high extent = red). Each dot represents a polymer formulation. SHAP dependency plots values across (C) P8 and P1, and (D) P5 and P1 relating to transfection performance. The *x*-axis represents the P8 and P5 content, respectively. The color bar corresponds to the P1 content (where low extent = blue; moderate extent = purple; high extent = red).

The importance of different components on overall PAE transfection performance was first studied, and the global importance factor of each component is illustrated in Figure 5A. From that, a clear influence law can be observed: the polymer component with the lowest MW (P1) exhibited the most significant influence on the transfection efficiency. For the other components P2–P8, their importance was enhanced with their MW. To further investigate whether a particular component affects the transfection in a positive or negative way, the SHAP summary plot that orders features (i.e., components P1–P8) based on their importance is shown in Figure 5B, with positive and negative impacts reflected by positive and negative SHAP values respectively). However, in contrast, as can be clearly seen, for most other components, when component content was too high it hindered the overall GFP expression (i.e., most red dots fell into the range where the SHAP values <0). For the high MW components P7 and P8, a moderate content is more favorable to transfection (i.e., the color of dots that appear in the position where the SHAP values are positive and relatively high is purple). Moreover, from Figure 5A and Figure 5B, it is interesting to note that component P6, which exhibited the best transfection efficiency when used alone (Figure 2A), showed less importance and negative correlation on the PAE transfection performance. This indicated that it is the synergistic interaction among different components which determines the ultimate PAE transfection capability.

For example, Figure 5C and D displays the possible interaction between the most important component P1 with P8 and P5 respectively. Figure 5C shows that when the SHAP value for P8 reached the highest positive value (i.e., the most favorable contribution to transfection, the P8 content falls between 5 and 10 on the *x*-axis), the content of P1 is relatively high or moderate (represented by the red or purple dots). This demonstrates that an appropriate combination of high MW P8 and low MW P1 work together to enhance the transfection performance. However, this synergistic behavior is different between the less important component P5 with P1. As shown in Figure 5D, when the SHAP value for P5 is positive and relatively high, the P1 content is mostly low (represented by the blue dots). The above results and analysis provided new insights into the transfection mechanism of polymeric vectors. They show how the different components within one polymer (high and low MW components) work together to synergistically affect the ultimate transfection performance and indicate that an appropriate combination ratio of different components (i.e., PCD) is essential for high transfection performance.

### Can High-performance Polymer Vectors be Designed by Optimizing PCD?

**Figure 6:**
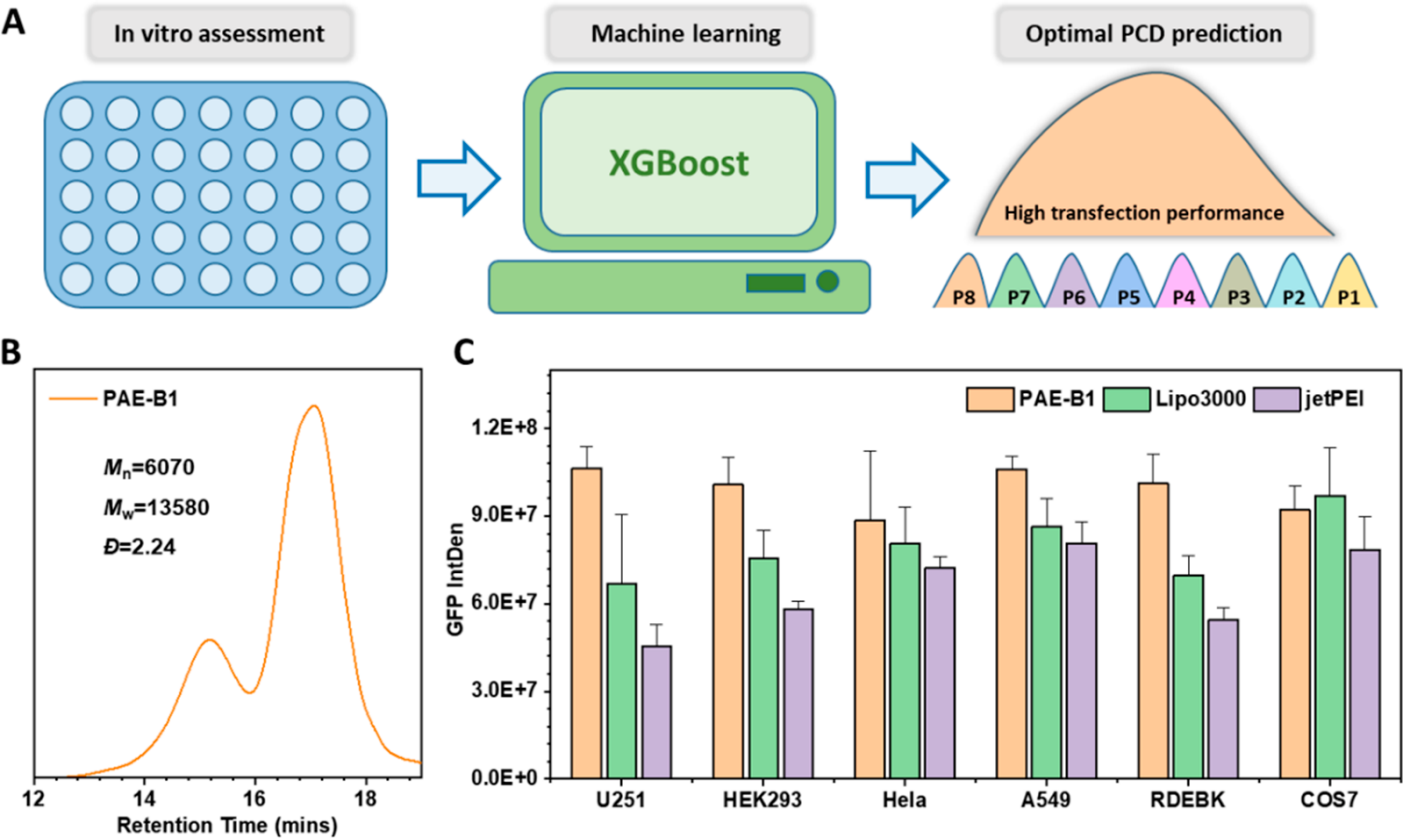
(A) Schematic illustration of the application of machine learning in optimizing the PCD of PAE polymers for better gene transfection. The trained XGBoost model was used to evaluate the transfection performance of 9 × 10^5^ PAEs with different PCDs. (B) SEC traces and polymer components of PAE-B1, which were selected according to the AI-predicted Top 1% rank of transfection performance. Its component ratio from P1 to P8 is 10:0:0:0:0:8:12:10. (C) Transfection results of PAE-B1 and commercial reagents based polyplex with different cells after 48 h at polymer/DNA w/w ratios of 40:1. The GFP expression in different cells were normalized to the highest expression in U251-MG cells.

Based on the above understanding of the effect of PCD on transfection performance, AI was further applied here to optimize polymer composition by adjusting the PCDs targeting high transfection efficiency. First, a library of polymers (9 × 10^5^) with different PCDs (i.e., different weight ratios of P1–P8) was computer generated. Subsequently, as illustrated in Figure 6A, the trained XGBoost model was applied to evaluate the transfection efficiency of polymers composed of different ratios of polymer components. According to the predicted results, these polymers were reordered according to their transfection efficiency (from high to low) to easily identify which polymers are optimal for gene delivery. Under this prediction guidance, polymer B1, which fell within the top 1% of the transfection order list, was selected and experimentally prepared (Figure 6B). It should, in principle, exhibit high transfection performance. The transfection in U251-MG cells verified that PAE-B1 exhibited higher gene delivery efficiency than original PAE-L1 and the best component alone (Figure S10). Subsequently, an *in vitro* assessment of the transfection performance of PAE-B1 was carried out using different cells. As predicted, the GFP expression of most cells after transfection by the AI-optimized PAE-B1 exceeded that of the commercial reagents jetPEI and Lipo3000 (Figure 6C). Notably, U251 cells demonstrated significantly higher transfection efficiency with the PAE-B1-based polyplex, exhibiting approximately 1.5 times GFP expression than Lipo3000 and 2 times that of jetPEI. The high transfection capability of PAE-B1 was also confirmed by delivering the Gaussia Princeps luciferase plasmid (Figure S11).

## Conclusion

In this work, the effect of polymer components and their distribution (PCD) on gene delivery performance was investigated for the first time via a combination of experiments and AI analysis. It is demonstrated that the PCD has an important effect on the overall transfection performance, which is a synergistic effect of the appropriate combination of different components within a polymer (high MW and low MW components). Moreover, a trained machine learning model successfully guided the preparation of a series of high-efficiency polymer vectors that far surpassed commercial reagents jetPEI and Lipo3000. This work provides novel insights into the structure–property relationship of PAEs from a new perspective, which will inspire new thinking for breaking the bottleneck in future high-efficiency polymer vector design.

## Supporting Information Available

Detailed experimental procedures, machine learning methods, NMR spectra, SEC traces measurements, cytotoxicity assay, transfection results, degradation analyses, and reconstructed polymer information (PDF)
