# Supplementary material for: Artificial Intelligence (AI)-Aided Structure Optimization for Enhanced Gene Delivery: The Effect of the Polymer Component Distribution (PCD)
Source: ACS Appl Mater Interfaces. 2023 Jul 21;15(30):36667–75. doi: 10.1021/acsami.3c05010 (PMC10401567; doi:10.1021/acsami.3c05010)
Supplement: Supplementary file 1 — am3c05010_si_001.pdf [file am3c05010_si_001.pdf]

Supporting Information

Artificial Intelligence (AI)-aided Structure Optimization for Enhanced Gene Delivery: The Effect of Polymer Component Distribution (PCD)

Yinghao Li,[†,‡,§] *Zhonglei He,**[‡,§] *Sigen A,*[‡,⊥] *Xianqing Wang,*[‡] *Zishan Li,*[‡] *Melissa Johnson,*[‡] *Ruth Foley,*[‡,§] *Irene Lara Sáez,*[‡] *Jing Lyu,**[‡] *and Wenxin Wang**[†,‡]

[†] Research and Clinical Translation Center of Gene Medicine and Tissue Engineering, School of Public Health, Anhui University of Science and Technology, Huainan, China

[‡] Charles Institute of Dermatology, School of Medicine, University College Dublin, Dublin 4, Ireland

[§] Branca Bunús Ltd, NovaUCD Belfield Innovation Centre, Ireland

[⊥] School of Medicine, Anhui University of Science and Technology, Huainan, China

KEYWORDS: gene delivery vectors, poly(β-amino ester), polymer component distribution (PCD), pDNA delivery, machine learning, artificial intelligence

1. Materials

1,4-butanediol diacrylate (BDA), 5-amino-1-pentanol (S5), were purchased from Sigma-Aldrich. 1-(3-aminopropyl)-4-methylpiperazine (E7) was purchased from Fisher Scientific. All amines used in termination were purchased from Sigma-Aldrich. Lithium bromide (LiBr) for SEC measurements was purchased from Sigma-Aldrich. Dimethyl sulfoxide (DMSO), dimethylformamide (DMF), acetone and diethyl ether were purchased from Fisher Scientific. Deuterated chloroform ($CDCl_3$) was purchased from Sigma-Aldrich. Hank's balanced salt solution, and alamarBlue Assay Kit purchased from Sigma and Invitrogen, respectively. Lipofectamine 3000 transfection reagent was purchased from Bio-sci. JetPEI were purchased from Polyplus Transfection, Illkirch-Graffenstaden, Strasbourg, France. Sodium acetate (Sigma) was diluted to 0.025 M prior to use. Picogreen was purchased from Life Technologies. Cell culture Dulbecco's modified Eagle Medium (DMEM) was purchased from Sigma. Fetal bovine serum (FBS, Gibco, was filtered through 0.2 μm filters before use. gWiz-GFP commercial plasmid was obtained from Aldevron, Fargo, ND, USA. Gaussia Princeps luciferase plasmid was obtained from New England Biolabs, London, UK. BioLux™ Gaussia Luciferase Assay Kit was obtained from New England Biolabs, Dublin, Ireland.

2. Experimental Method and Characterization Procedures

Synthesis procedure for Polymer L1

Polymer L1 was synthesized through a facile Michael addition reaction. Typically, BDA (7.92 g), and S5 (3.44 g) were dissolved in DMSO (50% w/v). The solution was bubbled under argon for 15 mins and then the reaction occurred at 90 ℃. The reaction was stopped in ~96 hours when $M_{w,SEC}$ was approaching target molecular weight by adding excessive BDA (7.92 g). Then

the reaction proceeded further for another 8 hours to eliminate the amine residue. Then the reaction was diluted into 10% w/v with DMSO and endcap with E7 at room temperature for 48 hours. Polymers were then precipitated into diethyl ether and dried under a vacuum before being stored at −20 ˚C. Agilent 1260 Infinite gel permeation chromatography (GPC/SEC) and nuclear magnetic resonance (NMR) were used to monitor the reaction.

Polyplex preparation

Generally, the polymers were initially dissolved in DMSO to stock solutions (100 mg/mL), and then the stock solutions were further diluted with 25 mM sodium acetate buffer according to the w/w ratio. DNA was diluted to 0.1 mg/mL with sodium acetate buffer. The polymer solutions were added into the DNA solution, vortexed for 10 s, and allowed to stand for 15 min.

Picogreen Assays

The polyplexes were prepared as described above. 2μg of DNA was used for each sample preparation. Then, 60 μL of Picogreen solution, which was prepared according to supplier's instructions, was added and allowed to incubate for another 5 min. In a 96-well plate, 30 μL of the polyplex solution was added to 200 μL of medium (without serum) or water. Fluorescence measurements were carried out with a plate reader with excitation at 490 nm and emission at 535 nm.

Size and Zeta Potential of Polyplexes

The polyplexes were prepared as described above. After that, the sizes and zeta potentials of polyplexes were measured with a Malvern Panalytical Zetasizer (ZTS1240). All the measurements were performed in triplicate.

Gel Electrophoresis

For DNA condensation study, 0.5 μg of DNA was used for each sample preparation. Polyplexes were first prepared as described above. Then polyplexes were loaded into the wells in agarose gel (1%) containing SYBR Safe DNA stain. Electrophoresis was carried out at 110 mV for 40 min. For DNA protection study, polyplexes were prepared as above, and then DNase I was added under the concentration of 10 U DNase / 1 μg DNA (+). Naked NDA with and without DNase were used as control. Polyplexes were incubated at 37 °C for 15 min. After that polyplexes were subjected to gel electrophoresis as mentioned above.

Cell Culture

Glioblastoma cells (U251-MG), human embryonic kidney cells (HEK293), Hela cells, human lung carcinoma cells (A549) and monkey kidney fibroblast-like cells (COS7) were cultured in Dulbecco's modified Eagle Medium high glucose (DMEM-HG), (Sigma-Aldrich, Dublin, Ireland) containing 10% fetal bovine serum (FBS). Immortalized primary human RDEB keratinocytes (RDEBK) were kindly provided by Dr. F. Larcher (Centro de Investigaciones Energéticas, Medioambientales y Tecnológicas-CIEMAT, Madrid, Spain), and cultured in the same DMEM-HG full culture medium. All cell lines were cultured at 37 ˚C with 5% CO_2 in a humidified incubator, using standard cell culture techniques.

Cytotoxicity Assessment (alamarBlue assay)

To perform the alamarBlue assay, cell supernatants were removed, and cells were washed with HBSS, followed by the addition of 10% alamarBlue reagent in HBSS. Living, proliferating cells maintain a reducing environment within the cytosol of the cell, converting the non-fluorescent ingredient resazurin in alamarBlue to the highly fluorescent compound resorufin. This reduction results in a colour change from blue to light red and allows for the quantitative

measurement of cell viability based on the increase in overall fluorescence and color of the media. The alamarBlue solution from each well was transferred to a fresh flat-bottomed 96-well plate for fluorescence measurements at 590 nm. Control cells without any treatment were used to normalize the fluorescence values and plotted as 100% viable.

Polyplex cellular uptake

GFP DNA was labelled with a Cy3 (a red fluorescent dye) labelling kit as per the recommended protocol. In 96-well plates, HEK cells were seeded. Gene transfection was conducted as above with 0.5 μg of DNA per well. After 4 hours, the medium was removed, and cells were fixed with 4% paraformaldehyde after washing with PBS three times. Next, the cells were permeabilized with 0.1% Triton X-100 and stained with DAPI, followed by visualization under a fluorescence microscope (Olympus IX81).

Cell transfection

Cells were transfected with polyplexes prepared as described above, mixed with the cell culture medium, and added to cells at DNA concentration of 5 μg/mL. Expression of the GFP reporter gene green fluorescent protein (GFP) was visualised 48 h after transfection using an Olympus IX81 fluorescence microscope. The intensity of GFP fluorescence was analysed and semi-quantified using the ImageJ software (NIH, Bethesda, MD, USA).

Size exclusion chromatography (SEC)

Number average molecular weight ($M_{n,SEC}$), weight average molecular weight ($M_{w,SEC}$), and polydispersity index (Đ) of polymers were determined by SEC equipped with a refractive index detector (RI), a viscometer detector (VS DP) and a dual angle light scattering detector (LS 15◦ and LS 90◦). To monitor the molecular weight of polymers during the polymerization process,

20 μL of the reaction mixture was collected at different time points, diluted with 1 mL of DMF, filtered through a 0.2 μm filter and then measured by SEC. The columns (PolarGel-M, Edinburgh, UK, 7.5 mm × 300 mm, two in series) were eluted with DMF and 0.1% LiBr at a flow rate of 1 mL/min at 60 ◦C. Columns were calibrated with linear poly(methyl methacrylate) (PMMA) standards.

Nuclear Magnetic Resonance (NMR)

The chemical structure and composition of polymers were confirmed with one- and two-dimensional NMR spectra of ^{1}H-NMR, ^{1}H, ^{1}H-COSY, ^{13}C, ^{1}H-HSQC, ^{13}C, ^{1}H-HSQC, ^{1}H, ^{1}H-TOCSY, and ^{13}C-NMR. Polymer samples were dissolved in $CDCl_3$. Measurements were carried out on a Varian Inova 400 MHz spectrometer (Edinburgh, UK). To monitor the reaction extent during the polymerization process, 100 μL of the reaction mixture was collected at different time points, diluted with 800 μL of deuterated solvent and then measured by NMR.

3. Machine Learning Method Description

Data Sets

In this study, the weight ratios of each component in different polymers (within a specific range of molecular weights for successful transfection: 2,000-30,000 Da) were used as input feature. For instance, in the case of Polymer-1 mentioned in Table S1, at a polymer/DNA weight ratio of 40:1 for transfection, the weight ratio of P1 to P8 is 2:6:8:8:7:4:3:2. Thus, in this particular sample, the input values would be (2, 6, 8, 8, 7, 4, 3, 2). The weight ratio of each component can vary from 0 to ∞, and the sum of the weight ratios from P1 to P8 should equal the polymer/DNA weight ratio (e.g., 40 in the case of Polymer-1). The corresponding output is the transfection efficiency, which is measured by the GFP expression and is represented in

logarithmic form for each sample.

Machine Learning Models

In this study, we employed six machine learning models to develop and validate models for optimizing the Polymer Component Distribution (PCD) of polymer vectors. The models used were Support Vector Machine (SVM), k-Nearest Neighbors (KNN), Decision Tree (DT), Extreme Tree (ET), Random Forest (RF), and eXtreme Gradient Boosting (XGBoost).

Support Vector Machine (SVM) is a supervised learning model used for regression tasks. It finds an optimal hyperplane that best fits the data points and maximizes the margin between the predicted values and the actual values. For regression tasks, SVM regression also follows the idea of maximum margin classification. SVM regression allows an error of ε between the true value and the predicted value, decision function following:

$$l_\varepsilon(w,b,x_i,y_i) = \begin{cases} 0, |f(x_i) - y_i| \leq \varepsilon \\ |f(x_i) - y_i| - \varepsilon, |f(x_i) - y_i| \geq \varepsilon \end{cases} \quad (1)$$

In this work, the Radial Basis Function (RBF) was used as kernel.

k-Nearest Neighbors (KNN) is a non-parametric supervised learning algorithm used for regression tasks. It predicts the value of a data point by averaging the values of its k nearest neighbors. For regression in KNN, the predicted value is typically the average of the k nearest neighbors' target values. A variety of formulas can measure the distance in the KNN algorithm, such as Euclidean distance (2), cosine distance (3) and Manhattan distance (4). $\boldsymbol{o}$ and $\boldsymbol{s}$ represent two samples, $\boldsymbol{o} = \boldsymbol{x}_0$ and $\boldsymbol{s} = \boldsymbol{x}_e$. Assuming the distance between sample $\boldsymbol{o}$ and $\boldsymbol{s}$ is d, the expression of Euclidean distance (2), cosine distance (3) and Manhattan distance (4) are shown below:

$$d(\boldsymbol{o},\boldsymbol{s}) = \sqrt{(\boldsymbol{x}_0 - \boldsymbol{x}_e)^2} \quad (2)$$

$$d(\boldsymbol{o},\boldsymbol{s}) = \frac{\boldsymbol{x}_0\boldsymbol{x}_e}{\|\boldsymbol{x}_0\|\|\boldsymbol{x}_e\|} \tag{3}$$

$$d(\boldsymbol{o},\boldsymbol{s}) = |\boldsymbol{x}_0 - \boldsymbol{x}_e| \tag{4}$$

In this work, the number of neighbors is set as 5.

Decision Tree (DT) is an interpretable supervised learning model that constructs a tree-like structure to make predictions based on simple decision rules learned from the data features. Decision trees make decisions based on a sequence of if-else conditions, where internal nodes represent feature tests, and leaf nodes represent prediction values. Taking the splitting process of a node in the decision tree algorithm model as an example to discuss the algorithm principle, the input data of the decision tree model can be formally expressed as $I = \{(x_i,y_i), (i = 1, 2,...,N),x_i \in R^m\}$, where x_i is m dimensional feature vector, y_i is the label value, N is the number of samples, and the expression of the decision tree model prediction value *f*(x) is shown in formula:

$$f(x) = \sum_{k-1}^{K} d_k C(x \in I_k) \tag{5}$$

C represents a constant, *K* represents the total number of areas that the decision tree model divides the input feature space into, *I* represents the input data sample, *k* represents the unit number that divides the input space into I_1,..., I_k,..., I_K, and d_k is the average prediction value of division units.

In this work, the max depth is set as 8.

Extreme Tree (ET), also known as Extremely Randomized Trees, is an ensemble learning method that builds a collection of uncorrelated decision trees. It introduces additional randomness during tree construction, reducing overfitting. Similar to Decision Trees, Extreme Trees use if-else conditions to make decisions, but during tree construction, random thresholds

are chosen instead of optimal ones, increasing the diversity among the trees.

In this work, the max depth is set as 8.

Random Forest (RF) is an ensemble learning method that constructs multiple decision trees and combines their predictions through averaging. It improves predictive accuracy and effectively handles high-dimensional data. In a Random Forest, predictions are aggregated by combining the predictions of individual decision trees. The predicted value in a Random Forest regression is the average of the predicted values of individual decision trees. Taking a splitting process of the decision tree model as an example, assuming that the sample data before splitting can be $I = \{(x_i,y_i), (i = 1, 2,...,N),x_i \in R^m\}$, then the regression error function can be expressed as the formula:

$$E(I) = \frac{1}{N}\sum_{i=1}^{N}(y_i - f(x_i))^2 \quad (6)$$

eXtreme Gradient Boosting (XGBoost) is an optimized implementation of gradient boosting, a powerful ensemble learning technique. It builds multiple weak learners, typically decision trees, in a sequential manner, focusing on the samples that were poorly predicted in previous iterations. XGBoost combines weak learners by using a weighted sum of their predictions. It minimizes a loss function by iteratively adding models to the ensemble, with each new model correcting the errors made by the previous models. XGBoost is a CART tree ensemble model, which uses the weighted sum of the predicted values of each tree in all trees as the prediction result. The predicted value of a model φ of K trees can be defined as:

$$\hat{y_i} = \varphi(x_i) = \sum_{k-1}^{K} f_k(x_i), f_k \epsilon F \quad (7)$$

By learning such a K tree model and making the error between the final predicted value and the real value of the model as small as possible, the objective function can be defined as:

$$min\ L(\varphi) = \sum_{i=1}^{n} l(y_i,\hat{y_i}) + \sum_{k=1}^{K} \Omega(f_k) \tag{8}$$

$$\Omega(f_k) = \gamma T + \frac{1}{2}\lambda \sum_{j-1}^{T} W_j^2 \tag{9}$$

The objective function consists of two parts: one part is the loss function l, which is used to measure the gap between the predicted score and the real score. The other part is the regularization term Ω. γ and λ, represent the penalty for the structural complexity of the CART decision tree, which can control the number of leaf nodes and limit the node score, preventing the model from overfitting the training data and loss prediction effect.

In this work, the max depth is set as 6.

Model Training and Evaluation

Then a ten-fold cross validation was conducted to compare the performance of different models. Predictive performance for the models was evaluated different metrics (Figure S9 and Table S3). Based on the evaluation, the final candidate model was selected. After that, the data was randomly split into a training set (80%) and a validation set (20%) and applied to the chosen model. The grid search (GS) technique was used to tune the hyperparameters of the model and used R^2 as evaluating index.

In this work, the selection of a supervised learning model for optimizing the polymer component ratio, instead of an optimization method like Bayesian optimization, is based on the following careful considerations: Firstly, Bayesian optimization typically requires running multiple experiments in parallel to iteratively update the model and search for the optimum. However, in the context of transfection, experimental results can be influenced by various factors, such as cell density, incubation time, passage number, etc., which makes it challenging to ensure consistent external conditions across different batches of experiments. Therefore,

utilizing machine learning that is based on data from the same batch offers a more viable option in terms of practicality and data integrity; Secondly, even though the Bayesian optimization techniques excel at efficiently exploring the parameter space to find an optimal solution, the objective of this work extends beyond solely locating the best ratio. We are also interested in uncovering the intricate relationship between PCD and transfection performance, aiming to gain a deeper understanding of the underlying factors that play a role in the gene delivery process. In this situation, a trade-off between uncovering the impact of PCD and finding the optimal PCD is necessary. By employing a supervised learning model, we prioritized exploiting the available data to construct a predictive model that can provide insights and predictions beyond the singular optimal solution. This approach enabled us to derive valuable insights about the relationship between PCD and transfection performance, contributing to a more comprehensive understanding of the system.

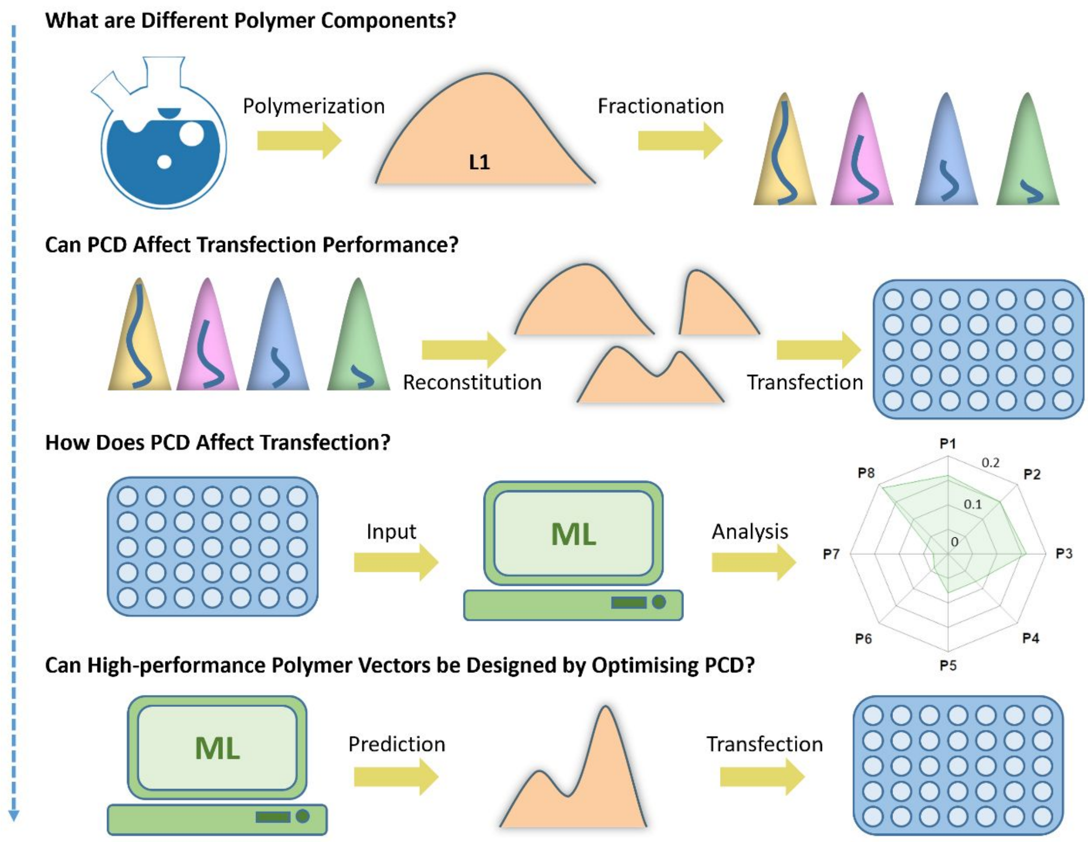

Scheme S1. Overall design program of this work.

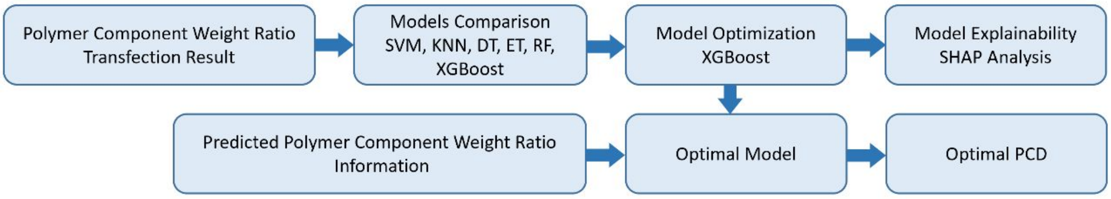

Scheme S2. Flow chart of this machine learning study.

4. Experiment Data

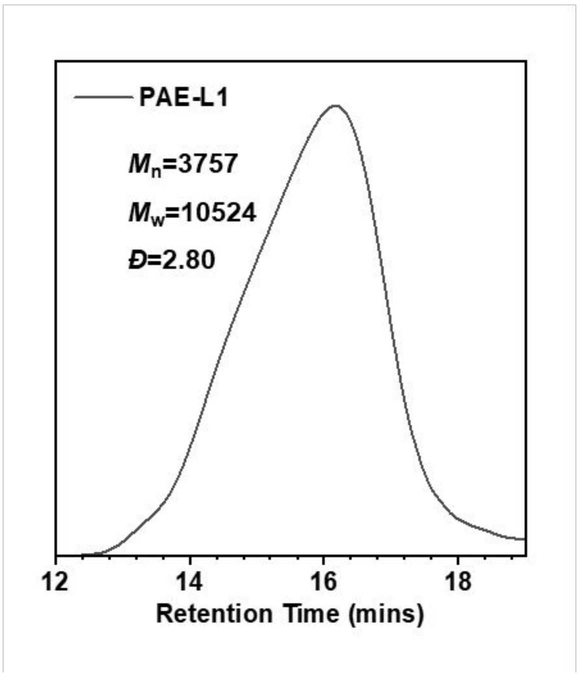

Figure S1. SEC characterization results of L1.

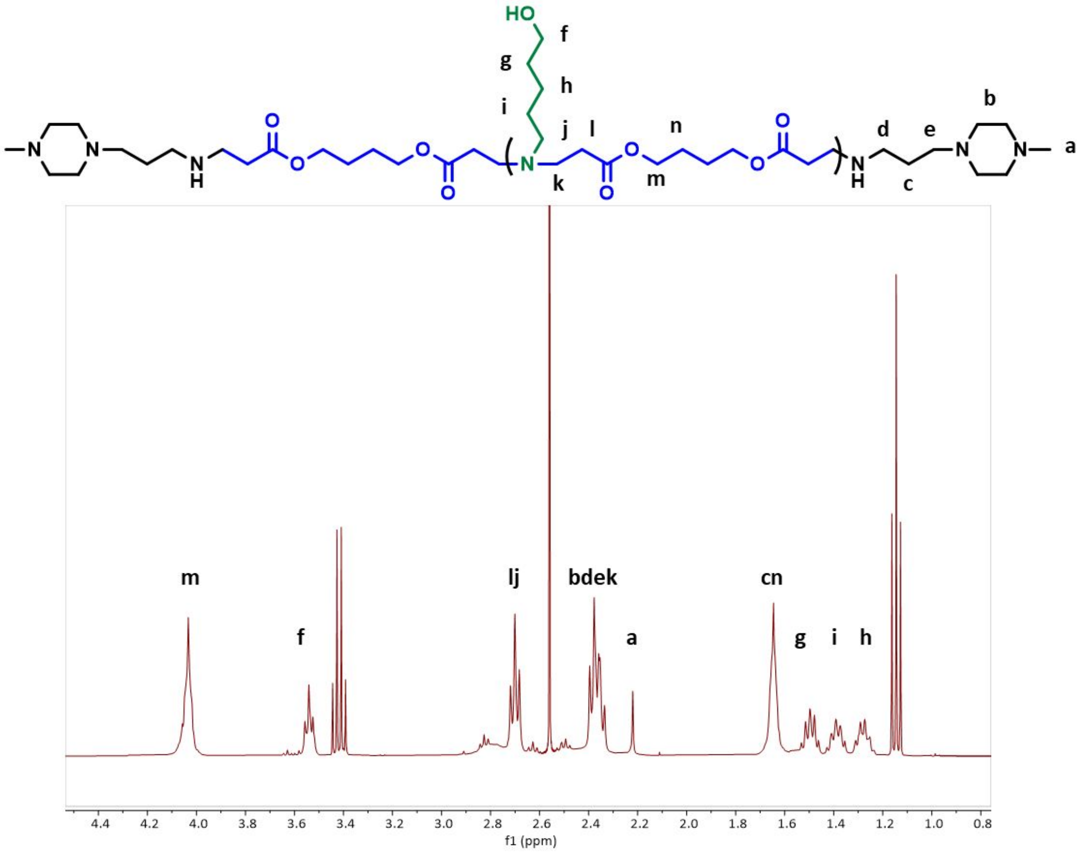

Figure S2. ^{1}H NMR spectra of PAE.

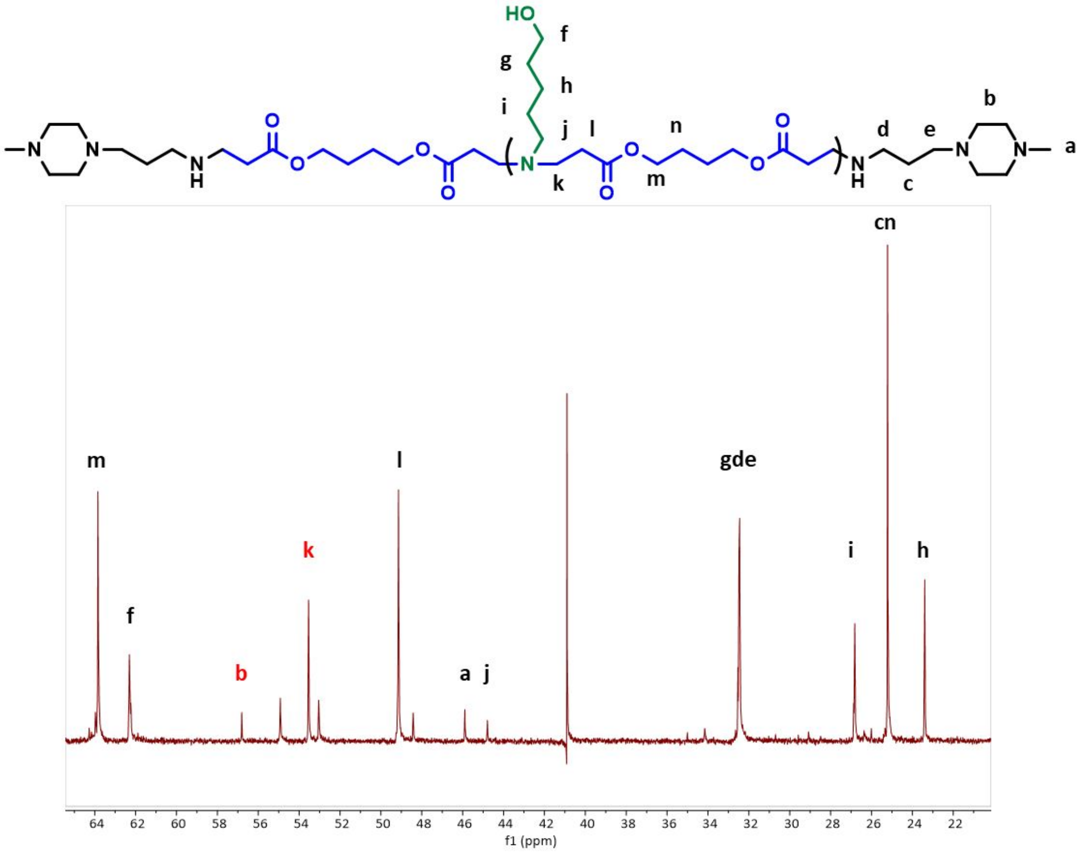

Figure S3. ^{13}C NMR spectra of PAE.

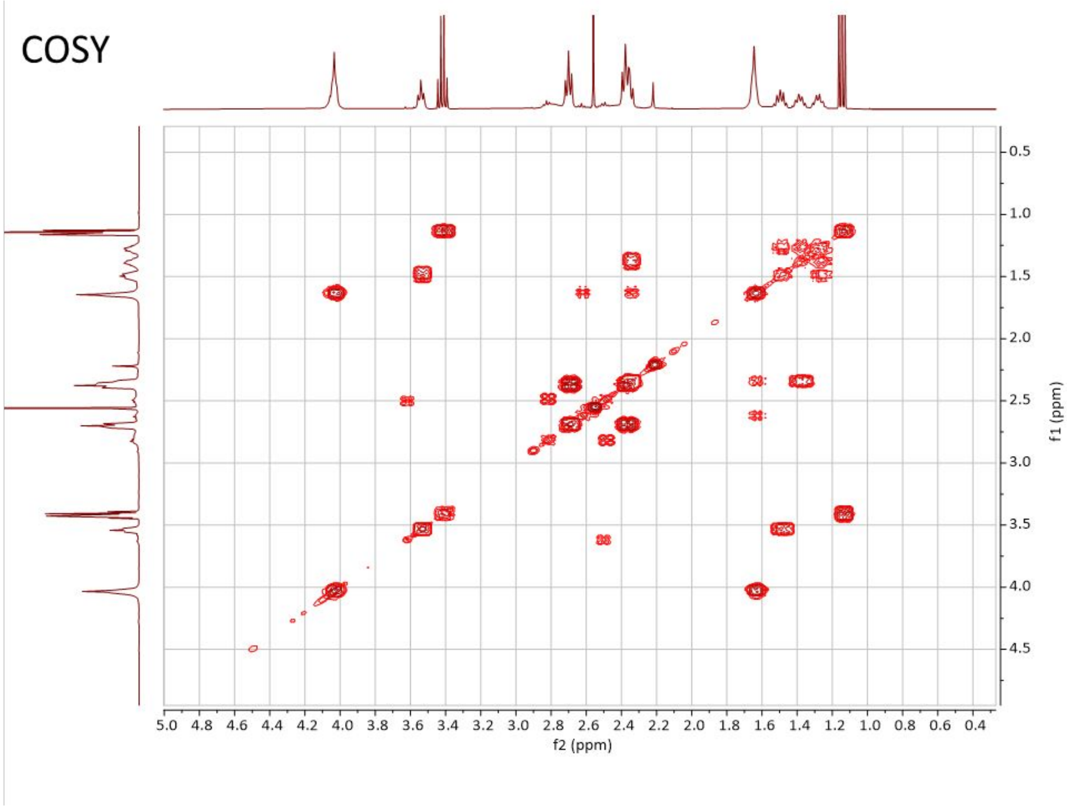

Figure S4. ^{1}H, ^{1}H -COSY spectra of the PAE.

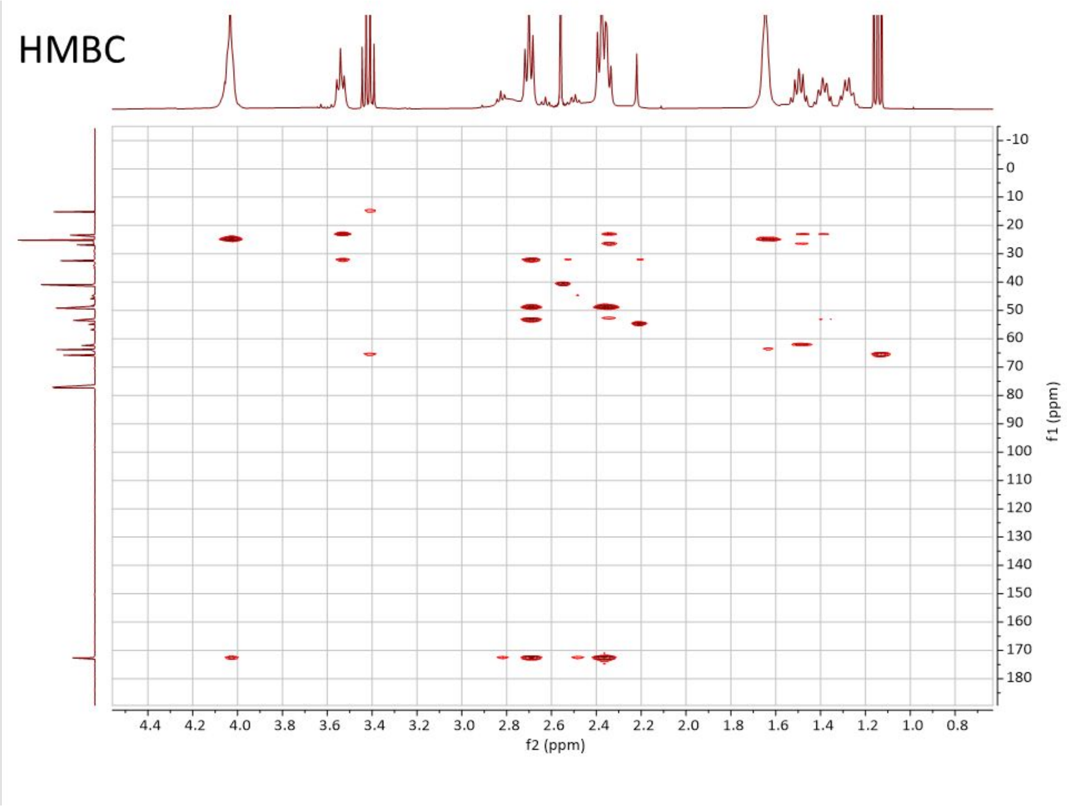

Figure S5. ^{13}C, ^{1}H -HMBC spectra of the PAE.

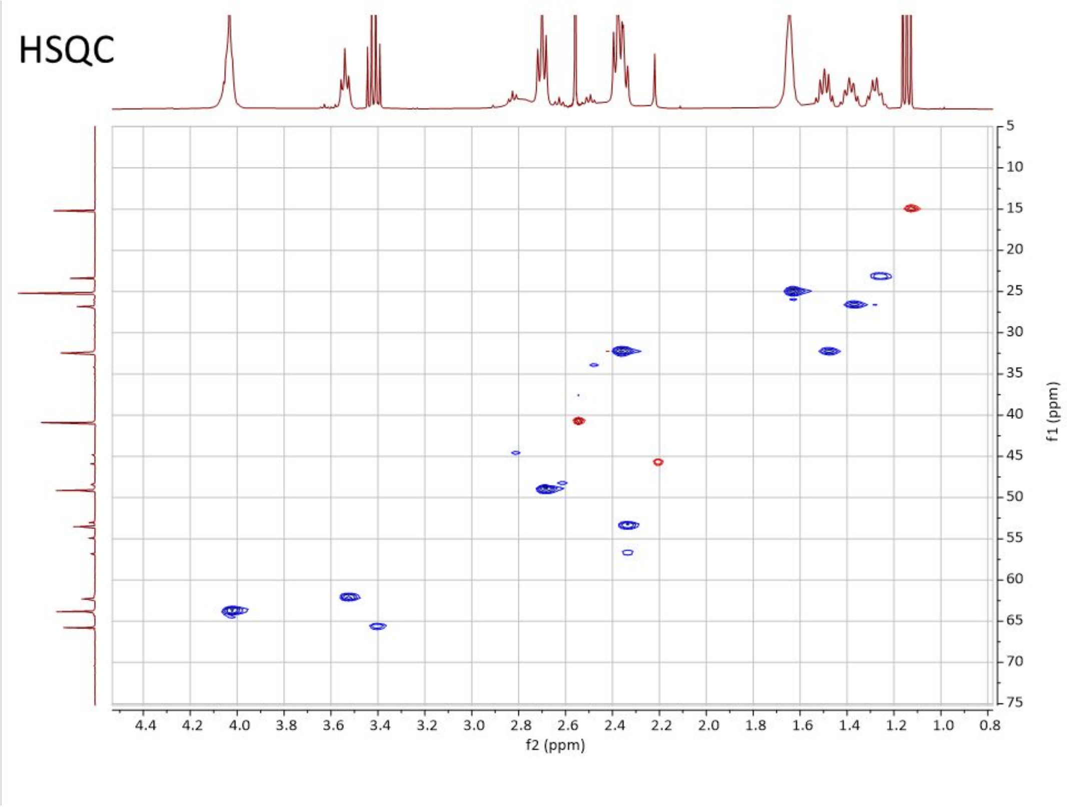

Figure S6. ^{13}C, ^{1}H -HSQC spectra of the PAE.

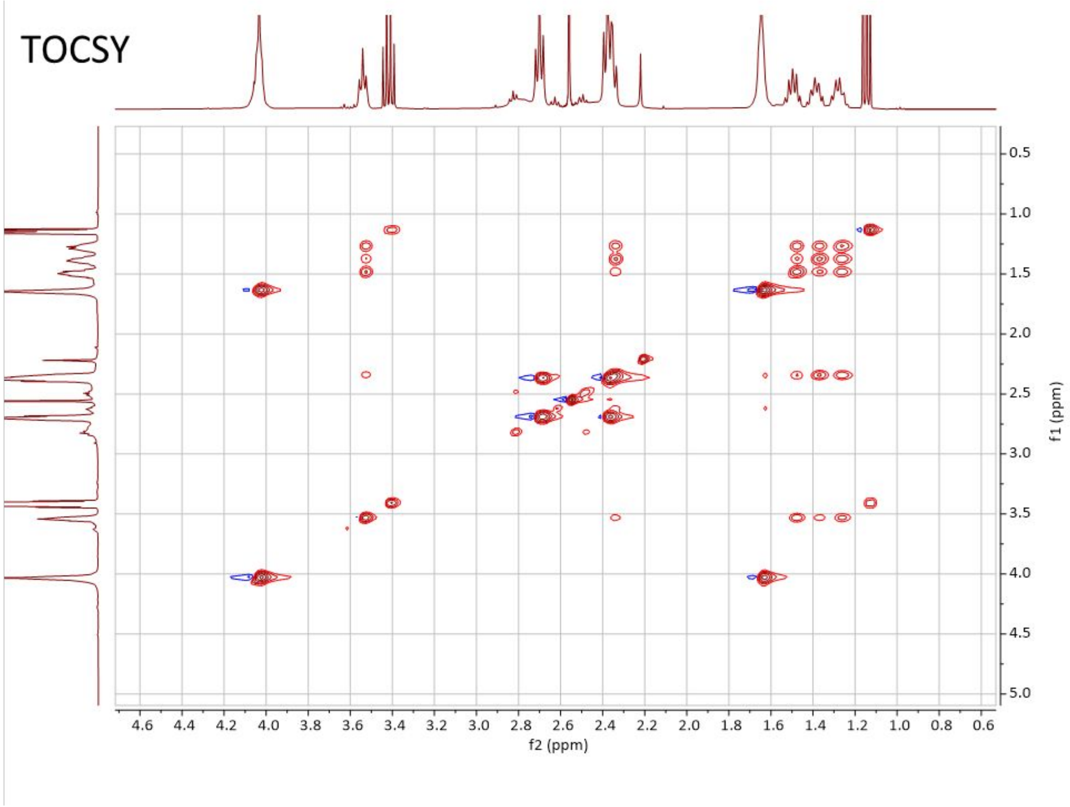

Figure S7. ^{1}H, ^{1}H -TOCSY spectra of the PAE.

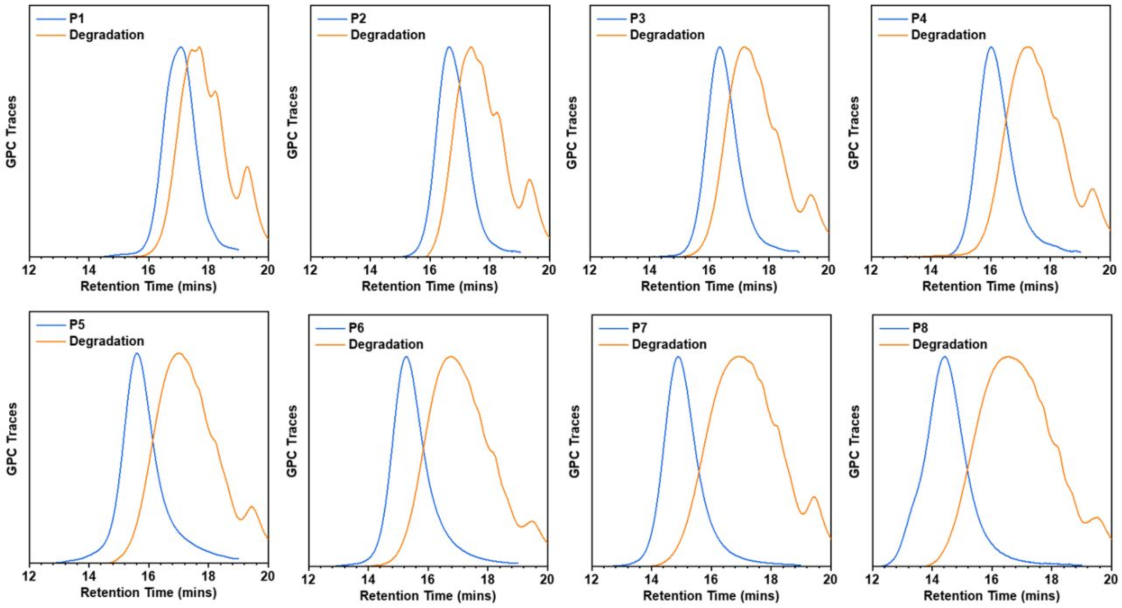

Figure S8. GPC Traces of P1 to P8 before and after degradation.

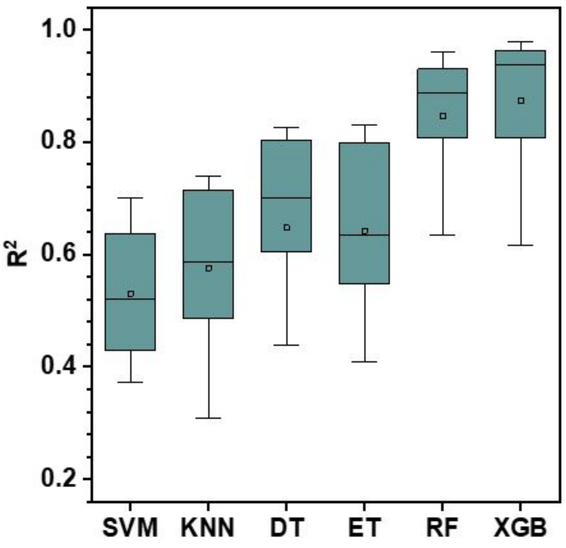

Figure S9. R^2 comparison of different algorithms in ten-fold cross validation.

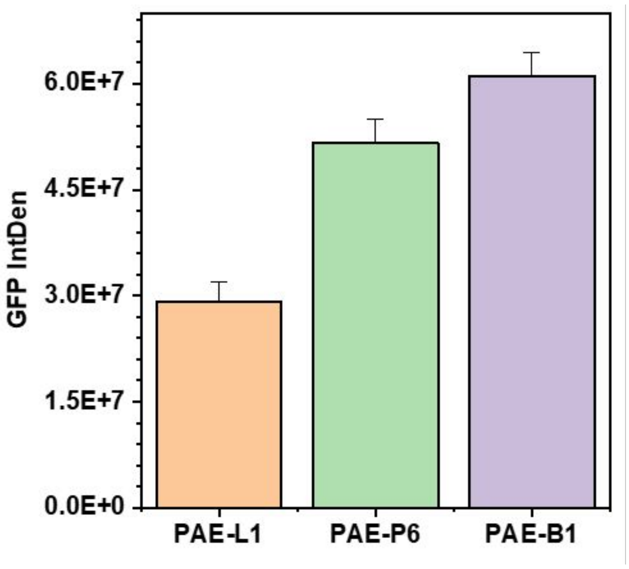

Figure S10. Transfection results of PAE-L1, P6, and B1- based polyplex in U251-MG cells 48 hours post transfection. Polymer/DNA w/w ratios of PAE-L1, P6, and B1 are 40:1, 60:1, 40:1, respectively.

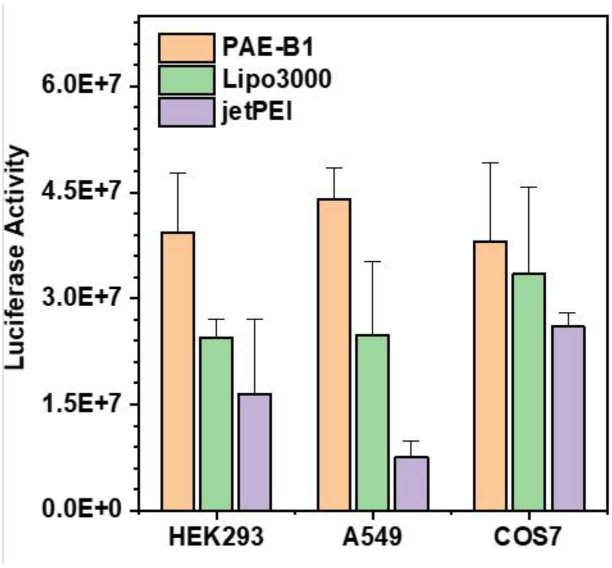

Figure S11. Luciferase activity of HEK293, A549, and COS7 cells after transfection with PAE-B1-based polyplex. The GFP expression in different cells were normalized to the highest expression in HEK293 cells.

Table S1. Weight ratios of P1 to P8 in polymer construction

Polymer	P8	P7	P6	P5	P4	P3	P2	P1
1	2	3	4	7	8	8	6	2
2	8	12	4	0	0	0	0	16
3	8	4	16	0	0	0	0	12
4	12	12	8	8	0	0	0	0
5	8	8	8	8	2	2	2	2
6	4	12	20	0	0	0	0	4
7	16	4	4	16	0	0	0	0
8	4	4	4	12	4	4	4	4
9	8	2	8	2	8	2	2	8
10	30	0	0	0	0	10	0	0
11	12	4	8	0	8	0	0	8
12	20	12	4	0	0	0	0	4
13	8	12	12	8	0	0	0	0
14	8	8	8	2	2	2	2	8
15	8	8	2	8	2	2	2	8
16	4	4	16	16	0	0	0	0
17	8	2	2	2	8	2	8	8
18	4	4	4	4	4	12	4	4
19	4	12	4	4	4	4	4	4

20	0	30	0	0	0	0	0	10
21	12	4	4	0	0	0	0	20
22	2	8	2	2	8	2	8	8
23	6	7	8	8	4	3	2	2
24	12	0	12	0	8	0	0	8
25	8	12	16	0	0	0	0	4
26	12	8	8	12	0	0	0	0
27	4	4	20	0	0	0	0	12
28	8	0	8	0	8	4	4	8
29	12	4	4	4	4	4	4	4
30	4	20	12	0	0	0	0	4
31	4	4	4	4	4	4	12	4
32	0	0	0	30	0	0	0	10
33	4	4	12	4	4	4	4	4
34	16	8	8	0	0	0	0	8
35	8	2	2	8	2	8	2	8
36	8	6	2	8	4	2	3	7
37	8	2	8	2	2	8	8	2
38	8	8	12	12	0	0	0	0
39	4	20	4	0	0	0	0	12
40	8	8	16	0	0	0	0	8
41	2	8	8	8	2	2	8	2

42	4	12	8	0	0	0	0	16
43	4	4	4	4	4	4	4	12
44	12	20	4	0	0	0	0	4
45	8	8	8	2	8	2	2	2
46	30	0	10	0	0	0	0	0
47	20	4	12	0	0	0	0	4
48	0	0	0	0	30	0	0	10
49	30	0	0	0	0	0	10	0
50	16	12	8	0	0	0	0	4
51	2	2	14	10	2	2	6	2
52	8	8	2	2	2	8	8	2
53	4	16	16	4	0	0	0	0
54	4	16	12	0	0	0	0	8
55	8	2	2	8	8	8	2	2
56	4	12	4	0	0	0	0	20
57	8	8	2	2	2	2	8	8
58	16	12	4	0	0	0	0	8
59	14	10	2	2	2	2	2	6
60	8	16	8	0	0	0	0	8
61	2	2	14	2	10	2	2	6
62	4	4	4	4	4	4	4	12
63	0	0	30	10	0	0	0	0

64	0	0	30	0	0	0	10	0
65	2	8	8	2	2	8	8	2
66	2	8	2	2	2	8	8	8
67	8	8	2	8	8	2	2	2
68	14	10	6	2	2	2	2	2
69	4	12	16	0	0	0	0	8
70	0	0	30	0	0	0	0	10
71	30	10	0	0	0	0	0	0
72	0	30	10	0	0	0	0	0
73	4	16	4	16	0	0	0	0
74	20	4	4	0	0	0	0	12
75	4	4	4	4	4	12	4	4
76	16	4	8	0	0	0	0	12
77	8	2	8	8	2	8	2	2
78	2	2	2	2	14	2	10	6
79	16	8	12	0	0	0	0	4
80	4	8	16	0	0	0	0	12
81	30	0	0	0	10	0	0	0
82	14	10	2	6	2	2	2	2
83	8	8	2	2	2	8	2	8
84	30	0	0	10	0	0	0	0
85	14	2	2	10	2	2	2	6

86	8	8	2	8	2	8	2	2
87	0	0	0	0	3	7	10	0
88	4	4	4	12	4	4	4	4
89	2	2	2	14	10	6	2	2
90	14	2	2	2	2	10	6	2
91	16	4	16	4	0	0	0	0
92	2	8	2	2	8	8	2	8
93	4	12	4	4	4	4	4	4
94	4	4	4	4	12	4	4	4
95	4	12	8	16	0	0	0	0
96	0	30	0	0	0	0	10	0
97	4	4	12	0	0	0	0	20
98	14	2	10	6	2	2	2	2
99	14	2	10	2	2	2	6	2
100	8	2	2	8	2	2	8	8
101	2	8	2	8	8	8	2	2
102	12	4	20	0	0	0	0	4
103	0	0	0	30	10	0	0	0
104	14	2	10	2	2	2	2	6
105	4	4	4	4	4	4	8	8
106	2	14	2	10	6	2	2	2
107	12	8	4	0	0	0	0	16

108	0	0	0	30	0	10	0	0
109	8	8	2	2	8	8	2	2
110	2	8	8	8	2	2	2	8
111	2	8	2	8	2	2	8	8
112	8	4	12	16	0	0	0	0
113	2	14	2	2	2	10	2	6
114	14	2	2	2	2	2	10	6
115	4	16	8	0	0	0	0	12
116	2	8	8	2	8	2	2	8
117	12	4	16	0	0	0	0	8
118	2	8	2	8	2	8	2	8
119	2	8	2	8	8	2	8	2
120	12	16	4	0	0	0	0	8
121	8	2	8	2	8	8	2	2
122	2	14	10	2	6	2	2	2
123	8	8	2	8	2	2	8	2
124	5	5	5	5	5	5	5	5
125	14	2	2	10	2	2	6	2
126	0	30	0	0	10	0	0	0
127	16	8	4	0	0	0	0	12
128	8	2	8	8	2	2	8	2
129	2	2	14	2	10	6	2	2

130	8	2	8	2	8	2	8	2
131	8	8	8	0	0	0	0	16
132	14	10	2	2	2	2	6	2
133	12	4	8	0	0	0	0	16
134	0	0	30	0	10	0	0	0
135	2	2	14	10	6	2	2	2
136	8	8	16	8	0	0	0	0
137	2	14	10	2	2	2	6	2
138	2	2	2	14	2	10	2	6
139	2	8	2	2	8	8	8	2
140	2	14	10	6	2	2	2	2
141	8	2	2	2	8	8	2	8
142	0	0	30	0	0	10	0	0
143	0	0	0	30	0	0	10	0
144	0	30	0	10	0	0	0	0
145	8	2	2	8	8	2	8	2
146	8	8	8	2	2	8	2	2
147	2	2	14	2	2	10	6	2
148	8	12	4	16	0	0	0	0
149	8	8	2	2	8	2	8	2
150	2	14	2	2	2	10	6	2
151	2	14	2	2	10	6	2	2

152	2	2	2	2	14	10	6	2
153	8	2	8	2	2	8	2	8
154	4	4	4	4	12	4	4	4
155	8	8	7	6	4	3	2	2
156	4	8	12	16	0	0	0	0
157	2	14	10	2	2	6	2	2
158	2	14	2	10	2	2	2	6
159	2	14	2	2	10	2	2	6
160	0	30	0	0	0	10	0	0
161	2	2	2	14	10	2	2	6
162	4	8	12	0	0	0	0	16
163	14	2	2	2	10	6	2	2
164	2	14	2	2	10	2	6	2
165	14	2	2	10	2	6	2	2
166	2	14	2	10	2	2	6	2
167	30	0	0	0	0	0	0	10
168	12	4	4	20	0	0	0	0
169	2	2	14	2	2	10	2	6
170	20	4	12	4	0	0	0	0
171	16	4	12	0	0	0	0	8
172	14	2	2	2	10	2	2	6
173	8	4	8	2	7	3	2	6

174	2	14	10	2	2	2	2	6
175	14	2	10	2	2	6	2	2
176	8	4	12	0	0	0	0	16
177	14	2	2	2	10	2	6	2
178	8	8	4	4	4	4	4	4
179	2	2	2	14	10	2	6	2
180	12	4	4	4	4	4	4	4
181	2	14	2	10	2	6	2	2
182	0	0	0	0	30	10	0	0
183	16	16	4	4	0	0	0	0
184	4	16	8	12	0	0	0	0
185	8	8	2	2	8	2	2	8
186	2	2	14	10	2	2	2	6
187	16	4	8	12	0	0	0	0
188	8	16	4	0	0	0	0	12
189	14	2	2	2	2	10	2	6
190	8	8	8	2	2	2	8	2
191	16	8	4	12	0	0	0	0
192	2	14	2	2	2	2	10	6
193	4	8	16	12	0	0	0	0
194	12	8	16	4	0	0	0	0
195	2	2	14	2	10	2	6	2

196	8	2	2	2	8	8	2	8
197	8	16	12	0	0	0	0	4
198	8	2	8	8	2	2	2	8
199	16	8	8	8	0	0	0	0
200	12	8	16	0	0	0	0	4
201	8	2	2	8	8	2	2	8
202	0	0	0	0	0	30	0	10
203	4	12	16	8	0	0	0	0
204	14	10	2	2	6	2	2	2
205	2	2	2	2	2	14	10	6
206	8	16	8	8	0	0	0	0
207	12	4	8	16	0	0	0	0
208	12	4	20	4	0	0	0	0
209	4	20	4	12	0	0	0	0
210	2	2	2	14	2	10	6	2
211	8	2	8	2	2	2	8	8
212	4	16	12	8	0	0	0	0
213	2	2	14	2	2	2	10	6
214	2	2	2	14	2	2	10	6
215	4	4	20	12	0	0	0	0
216	16	12	4	8	0	0	0	0
217	12	20	4	4	0	0	0	0

218	2	8	2	8	2	8	8	2
219	4	4	4	4	4	4	12	4
220	16	12	8	4	0	0	0	0
221	2	2	2	2	12	9	2	9
222	2	2	14	10	2	6	2	2
223	4	4	12	4	4	4	4	4
224	8	2	2	8	2	8	8	2
225	4	12	20	4	0	0	0	0
226	8	4	16	12	0	0	0	0
227	8	2	8	8	8	2	2	2
228	4	12	4	20	0	0	0	0
229	20	4	4	12	0	0	0	0
230	16	8	12	4	0	0	0	0
231	12	4	16	8	0	0	0	0
232	4	20	12	4	0	0	0	0
233	20	12	4	4	0	0	0	0
234	2	8	8	8	8	2	2	2
235	8	2	2	2	8	8	8	2
236	8	12	16	4	0	0	0	0
237	2	8	8	2	8	2	8	2
238	12	16	8	4	0	0	0	0
239	2	8	8	8	2	8	2	2

240	2	8	8	2	2	2	8	8
241	12	16	4	8	0	0	0	0
242	2	8	8	2	2	8	2	8
243	2	8	8	2	8	8	2	2
244	0	0	0	0	0	0	30	10
245	8	16	4	12	0	0	0	0
246	12	8	16	0	0	0	0	4
247	0	0	0	0	30	0	10	0
248	14	10	2	2	2	6	2	2
249	8	8	8	16	0	0	0	0
250	2	8	2	8	8	2	2	8
251	14	2	10	2	6	2	2	2
252	14	2	2	10	6	2	2	2
253	8	16	12	4	0	0	0	0
254	16	4	20	0	0	0	0	0
255	4	4	12	20	0	0	0	0
256	12	16	8	0	0	0	0	4

Table S2. SEC characterization of reconstituted polymer

Polymer	$M_{n,SEC}$ (Da)	$M_{w,SEC}$ (Da)	$Đ$
1	4635	6112	1.32
2	4509	11829	2.62
3	5154	10489	2.03
4	12214	15464	1.27
5	7317	11692	1.60
6	8220	10916	1.33
7	10786	15601	1.45
8	5092	7771	1.53
9	4923	9529	1.94
10	10732	22355	2.08
11	5866	12579	2.14
12	10669	19748	1.85
13	11254	13256	1.18
14	5470	11269	2.06
15	5257	11045	2.10
16	8799	8977	1.02
17	4127	8966	2.17
18	4565	7521	1.65
19	5600	9842	1.76
20	6084	12048	1.98

21	3869	11833	3.06
22	4051	6983	1.72
23	6666	10161	1.52
24	5732	11693	2.04
25	8721	13124	1.50
26	11015	14429	1.31
27	4975	8281	1.66
28	4468	8836	1.98
29	5795	12486	2.15
30	8793	12688	1.44
31	4188	7320	1.75
32	4326	4280	0.99
33	5362	8070	1.51
34	7123	16222	2.28
35	4533	9304	2.05
36	5139	10479	2.04
37	5030	9614	1.91
38	10229	12221	1.19
39	5401	11825	2.19
40	6479	11806	1.82
41	5367	7818	1.46
42	4371	9621	2.20

43	3877	7207	1.86
44	10024	17104	1.71
45	6963	11505	1.65
46	19061	23041	1.21
47	9837	17976	1.83
48	3760	3343	0.89
49	8491	22104	2.60
50	9929	17540	1.77
51	5504	6602	1.20
52	5185	10943	2.11
53	11504	12084	1.05
54	6519	11371	1.74
55	5512	9540	1.73
56	3781	9189	2.43
57	4505	10707	2.38
58	7330	17108	2.33
59	6557	15240	2.32
60	6830	13578	1.99
61	4987	6295	1.26
62	3877	7207	1.86
63	8534	6108	0.72
64	5878	5545	0.94

65	4918	7631	1.55
66	3893	6983	1.79
67	6621	11281	1.70
68	8562	15672	1.83
69	6355	10485	1.65
70	5152	5403	1.05
71	23499	25256	1.07
72	13318	13127	0.99
73	9827	11635	1.18
74	5778	17112	2.96
75	4565	7521	1.65
76	5554	14905	2.68
77	5987	9952	1.66
78	3812	5271	1.38
79	9553	16654	1.74
80	5075	9167	1.81
81	13087	22355	1.71
82	8214	15522	1.89
83	4825	10858	2.25
84	15425	22667	1.47
85	5871	13169	2.24
86	6208	11281	1.82

87	3488	3485	1.00
88	5092	7771	1.53
89	5104	6003	1.18
90	5452	12976	2.38
91	12841	16050	1.25
92	4309	7134	1.66
93	5600	9842	1.76
94	4862	7521	1.55
95	9458	10749	1.14
96	7123	12190	1.71
97	3671	7417	2.02
98	7712	13750	1.78
99	6629	13525	2.04
100	4249	9153	2.15
101	5378	7557	1.41
102	8649	13560	1.57
103	6029	4673	0.78
104	6233	13468	2.16
105	4027	7264	1.80
106	6526	9360	1.43
107	4571	13151	2.88
108	5476	4673	0.85

109	5951	11093	1.86
110	4983	7733	1.55
111	4169	7171	1.72
112	9374	11185	1.19
113	4949	8953	1.81
114	4702	12718	2.70
115	5288	10939	2.07
116	4816	7546	1.57
117	6608	13128	1.99
118	4442	7321	1.65
119	4983	7406	1.49
120	7172	15786	2.20
121	5748	9764	1.70
122	6976	9660	1.38
123	5687	11130	1.96
124	4832	8467	1.75
125	6221	13225	2.13
126	10097	12441	1.23
127	5679	15791	2.78
128	5501	9801	1.78
129	5522	6452	1.17
130	5298	9614	1.81

131	4430	10943	2.47
132	6997	15297	2.19
133	4490	12265	2.73
134	7766	5796	0.75
135	6056	6702	1.11
136	10774	12370	1.15
137	6254	9559	1.53
138	4371	5846	1.34
139	4594	7219	1.57
140	7209	9784	1.36
141	4395	9117	2.07
142	6872	5796	0.84
143	4826	4422	0.92
144	11435	12753	1.12
145	5098	9389	1.84
146	6507	11505	1.77
147	4893	6352	1.30
148	10127	12957	1.28
149	5471	10943	2.00
150	5196	9010	1.73
151	5910	9110	1.54
152	4290	5528	1.29

153	4691	9529	2.03
154	4862	7521	1.55
155	6990	11561	1.65
156	9117	9863	1.08
157	6664	9660	1.45
158	5575	9203	1.65
159	5301	8953	1.69
160	8635	12441	1.44
161	4643	5846	1.26
162	4297	8735	2.03
163	6244	13076	2.09
164	5585	9010	1.61
165	6627	13326	2.01
166	5890	9260	1.57
167	7056	21962	3.11
168	9557	13243	1.39
169	4674	6295	1.35
170	14105	18258	1.29
171	6928	15336	2.21
172	5568	12919	2.32
173	5407	10187	1.88
174	5900	9502	1.61

175	7092	13625	1.92
176	4353	10057	2.31
177	5882	12976	2.21
178	5696	11164	1.96
179	4860	5903	1.21
180	5795	12486	2.15
181	6252	9360	1.50
182	4600	3736	0.81
183	15155	18708	1.23
184	10329	11785	1.14
185	5072	10858	2.14
186	5228	6545	1.25
187	11394	15751	1.38
188	5491	13147	2.39
189	5181	12919	2.49
190	5937	11355	1.91
191	11933	16637	1.39
192	4510	8752	1.94
193	9547	10013	1.05
194	12362	14728	1.19
195	5237	6352	1.21
196	4395	9117	2.07

197	9033	14010	1.55
198	5098	9716	1.91
199	12681	16786	1.32
200	8956	14446	1.61
201	4750	9304	1.96
202	3162	3343	1.06
203	10435	11049	1.06
204	7913	15397	1.95
205	3540	5271	1.49
206	11780	14142	1.20
207	10031	13393	1.34
208	11785	13842	1.17
209	10770	12671	1.18
210	4562	5903	1.29
211	4387	9378	2.14
212	10885	11935	1.10
213	4281	6094	1.42
214	4025	5645	1.40
215	9199	9127	0.99
216	13352	17672	1.32
217	14492	17386	1.20
218	4745	7406	1.56

219	4188	7320	1.75
220	14296	17822	1.25
221	3808	5171	1.36
222	5819	6702	1.15
223	5362	8070	1.51
224	4849	9389	1.94
225	11003	11198	1.02
226	9830	11335	1.15
227	6370	9952	1.56
228	9036	10599	1.17
229	12378	17958	1.45
230	13530	16936	1.25
231	11136	13692	1.23
232	12054	12970	1.08
233	15881	20030	1.26
234	6192	7969	1.29
235	4691	9202	1.96
236	11918	13406	1.12
237	5174	7631	1.47
238	13705	16500	1.20
239	5829	7969	1.37
240	4302	7395	1.72

241	12835	16350	1.27
242	4593	7546	1.64
243	5602	7781	1.39
244	2564	2590	1.01
245	11131	13993	1.26
246	8956	14446	1.61
247	4133	3485	0.84
248	7514	15397	2.05
249	9736	12071	1.24
250	4650	7321	1.57
251	7446	13625	1.83
252	6935	13326	1.92
253	12509	14292	1.14
254	13712	16200	1.18
255	8432	8827	1.05
256	9641	16218	1.68

Table S3. Model performance of test set using different algorithms

Model	MSE	MAE	RMSE	MAPE	R^2
SVM	2.3439	0.7729	1.5310	0.0664	0.5927
KNN	1.8971	0.7663	1.3774	0.0618	0.6703
DT	1.5475	0.6516	1.2440	0.0467	0.7311
ET	1.6498	0.7674	1.2845	0.0565	0.7133
RF	0.6985	0.4146	0.8358	0.0320	0.8786
XGB	0.6660	0.3237	0.8161	0.0229	0.8843

MSE: Mean Squared Error, MAE: Mean Absolute Error, RMSE: Root Mean Squared Error, MAPE: Mean Absolute Percentage Error, R^2: Coefficient of Determination.

Table S4. Model performance of training set using different algorithms

Model	MSE	MAE	RMSE	MAPE	R^2
SVM	1.8032	0.6758	1.3428	0.0540	0.5897
KNN	0.9078	0.5480	0.9528	0.0405	0.7935
DT	1.4342	0.6572	1.1976	0.0466	0.6737
ET	1.3546	0.7023	1.1639	0.0501	0.6918
RF	0.1769	0.2324	0.4206	0.0160	0.9598
XGB	0.1473	0.1964	0.3838	0.0128	0.9665

MSE: Mean Squared Error, MAE: Mean Absolute Error, RMSE: Root Mean Squared Error, MAPE: Mean Absolute Percentage Error, R^2: Coefficient of Determination.
